# Bringing the Nature Futures Framework to life: creating a set of illustrative narratives of nature futures

**DOI:** 10.1007/s11625-023-01316-1

**Published:** 2023-05-04

**Authors:** América Paz Durán, Jan J. Kuiper, Ana Paula Dutra Aguiar, William W. L. Cheung, Mariteuw Chimère Diaw, Ghassen Halouani, Shizuka Hashimoto, Maria A. Gasalla, Garry D. Peterson, Machteld A. Schoolenberg, Rovshan Abbasov, Lilibeth A. Acosta, Dolors Armenteras, Federico Davila, Mekuria Argaw Denboba, Paula A. Harrison, Khaled Allam Harhash, Sylvia Karlsson-Vinkhuyzen, HyeJin Kim, Carolyn J. Lundquist, Brian W. Miller, Sana Okayasu, Ramon Pichs-Madruga, Jyothis Sathyapalan, Ali Kerem Saysel, Dandan Yu, Laura M. Pereira

**Affiliations:** 1 Instituto de Ciencias Ambientales y Evolutivas, Facultad de Ciencias, Universidad Austral de Chile, Valdivia, Chile; 2 Instituto de Ecología y Biodiversidad (IEB-Chile), Santiago, Chile; 3 Stockholm Resilience Centre, Stockholm University, Kräftriket 2B, 104 05 Stockholm, Sweden; 4 National Institute for Space Research (INPE), Av. dos Astronautas 1758, São José dos Campos, SP CEP: 12227-010 Brazil; 5 Institute for the Oceans and Fisheries, The University of British Columbia, Vancouver, Canada; 6 African Model Forests Network, BP 33678, Yaoundé, Cameroon; 7 African Model Forests Network, BP 2384, Dakar, Senegal; 8 IFREMER, Unité halieutique Manche‐Mer du Nord Ifremer, HMMN, 62200 Boulogne sur Mer, France; 9 Department of Ecosystem Studies, University of Tokyo, Tokyo, Japan; 10 Fisheries Ecosystems Laboratory (LabPesq), Oceanographic Institute, University of Sao Paulo, São Paulo, Brazil; 11 PBL Netherlands Environmental Assessment Agency, The Hague, The Netherlands; 12 Department of Geography and Environment, Khazar University, Baku, Azerbaijan; 13 Climate Action and Inclusive Development Department, Global Green Growth Institute (GGGI), Seoul, South Korea; 14 Departamento de Biologia, Universidad Nacional de Colombia, Bogota, Colombia; 15 Institute for Sustainable Futures, University of Technology Sydney, Sydney, Australia; 16 Center for Environmental Science, Addis Ababa University, Addis Ababa, Ethiopia; 17 UK Centre for Ecology and Hydrology, Bailrigg, Lancaster, UK; 18 Egyptian Environmental Affairs Agency, Ministry of Environment, Cairo, Egypt; 19 Public Administration and Policy Group, Wageningen University, Wageningen, The Netherlands; 20 German Centre for Integrative Biodiversity Research (iDiv) Halle-Jena-Leipzig, Leipzig, Germany; 21 Martin Luther University Halle-Wittenberg, Halle (Salle), Germany; 22 National Institute of Water and Atmospheric Research, Hamilton, New Zealand; 23 School of Environment, The University of Auckland, Auckland, New Zealand; 24 U.S. Geological Survey, North Central Climate Adaptation Science Center, Boulder, CO USA; 25 Centro de Investigaciones de la Economia Mundial (CIEM), La Habana, Cuba; 26 National Institute of Rural Development and Panchayati Raj Hyderabad, Hyderabad, India; 27 Institute of Environmental Sciences, Boğaziçi University, Istanbul, Turkey; 28 Nanjing Institute of Environmental Sciences (NIES), Ministry of Ecology and Environment (MEE) of China, 8 Jiangwangmiao Street, Nanjing, 210042 People’s Republic of China; 29 Global Change Institute, Wits University, Johannesburg, South Africa

**Keywords:** Biodiversity, IPBES, Nature values, NCP, Scenarios, Transformation

## Abstract

To halt further destruction of the biosphere, most people and societies around the globe need to transform their relationships with nature. The internationally agreed vision under the Convention of Biological Diversity—Living in harmony with nature—is that “By 2050, biodiversity is valued, conserved, restored and wisely used, maintaining ecosystem services, sustaining a healthy planet and delivering benefits essential for all people”. In this context, there are a variety of debates between alternative perspectives on how to achieve this vision. Yet, scenarios and models that are able to explore these debates in the context of “living in harmony with nature” have not been widely developed. To address this gap, the Nature Futures Framework has been developed to catalyse the development of new scenarios and models that embrace a plurality of perspectives on desirable futures for nature and people. In this paper, members of the IPBES task force on scenarios and models provide an example of how the Nature Futures Framework can be implemented for the development of illustrative narratives representing a diversity of desirable nature futures: information that can be used to assess and develop scenarios and models whilst acknowledging the underpinning value perspectives on nature. Here, the term *illustrative* reflects the multiple ways in which desired nature futures can be captured by these narratives. In addition, to explore the interdependence between narratives, and therefore their potential to be translated into scenarios and models, the six narratives developed here were assessed around three areas of the transformative change debate, specifically, (1) land sparing vs. land sharing, (2) Half Earth vs. Whole Earth conservation, and (3) green growth vs. post-growth economic development. The paper concludes with an assessment of how the Nature Futures Framework could be used to assist in developing and articulating transformative pathways towards desirable nature futures.

**Supplementary Information:**

The online version contains supplementary material available at https://doi.org/10.1007/s11625-023-01316-1.

## Introduction

What type of living world will exist in 50 or 100 years? How will human activities be intertwined with natural processes? Parties to the Convention on Biological Diversity (CBD) have agreed to move towards a world in which humanity is “Living in harmony with nature”, by 2050. There are many ways that people could plausibly live in harmony with nature; however, there exist only a limited number of scenarios that describe a desirable future for both nature and people, covering a relatively narrow range of possibilities and pathways (Leclère et al. 2020; Wyborn et al. 2020).

Popular discussion of environmental futures, in novels, TV series, and films, is dominated by dystopian visions of human ill-being and environmental degradation (Bennett et al. 2016; McPhearson et al. 2016; Berber 2018). This is understandable, because recent history has been characterised by the rapid unravelling of the fabric of life that supports humanity (Diaz 2022). However, shifting to a trajectory that reweaves the web of life will require transformative change (Diaz et al. 2019; IPBES 2019; Secretariat of the Convention on Biological Diversity 2020), and a key step to promote such change is the identification of pathways to reach a world that achieves the goal of “living in harmony with nature” (Wyborn et al. 2020; IPBES 2016; Shin et al. 2019).

It is important that alternative visions or pathways towards the goal of “living in harmony with nature” are considered, because people and organisations have multiple views of what such a world would look like, what values such a world should recognise and embrace, and what types of changes are needed to create such a world. Alternative views generate debates that range over many connected topics. For example, there is a well-established, and much criticised, debate within conservation as to whether land sparing (separation) or land sharing (integrating) of conservation and food production would maximise sustainability outcomes (Fischer et al. 2014; Loconto et al. 2020; Collas et al. 2023). A second debate has emerged over whether biodiversity conservation would benefit more from a ‘Half Earth’ approach that protects half the earth, including land and ocean, from human impact (Wilson 2016), and with a focus on justice for non-human species (Kopnina 2016) versus a ‘Whole Earth’ approach that argues that biodiversity would be better protected by addressing the main drivers of biodiversity loss (Büscher et al. 2017). Another debate focuses on the configuration of economies away from a growth-oriented paradigm as being pivotal in achieving biodiversity conservation targets (Moranta et al. 2022; Otero et al. 2020) and there has been a call to include post-growth scenarios in the analysis of different climate trajectories (Hickel et al. 2021). These are three examples of the most influential debates of which changes are needed for people to live in harmony with nature, as sought for under the CBD. A scenario framework to systematically assess alternative trajectories of nature needs to be able to include plural perspectives to enable a transparent debate on the value choices underlying each potential intervention.

While academic work on global futures does include futures in which humanity manages to address climate change and development challenges, few scenarios exist that describe a future in which global biodiversity targets are achieved (Pereira et al. 2020a, b; Schipper et al. 2020). Global assessment studies, such as the Millennium Ecosystem Assessment and the IPBES Global Assessment, have assessed how well different futures succeed in achieving at least a few key targets on biodiversity, but generally these studies indicate that even “sustainability scenarios” are unlikely to achieve global biodiversity targets (Sala et al. 2005; Shin et al. 2019; Schipper et al. 2020). Some target-seeking scenarios do exist, showing that biodiversity loss can be reversed if a portfolio of additional measures is incorporated, including increased conservation efforts and the critically important inclusion of measures tackling the drivers of biodiversity loss (Chai et al. 2019; Leclère et al. 2020). A greater diversity of pathways that articulate alternative nature-rich futures is needed to enable people and organisations to better imagine strategies, policies and actions that can achieve the CBD’s goals of living in harmony with nature.

### The Nature Futures Framework

The Intergovernmental Science-Policy Platform on Biodiversity and Ecosystem Services (IPBES) supports the development of new nature-oriented scenarios. The IPBES Plenary at its 7th session (2019) established a task force on scenarios and models, whose role includes catalysing the further development of scenarios and models by the broader scientific community for future IPBES assessments. This work builds on the IPBES Methodological Assessment of Scenarios and Models of Biodiversity and Ecosystem Services that identified a range of knowledge gaps and challenges (IPBES 2016). Following decision IPBES-4/1 by the IPBES Plenary, the task force began a participatory process to catalyse the filling of knowledge gaps and development of desired nature futures. This led to the development of the Nature Futures Framework, a flexible tool to support the development of scenarios and models of desirable futures for people, nature and Mother Earth,^1^ described in Pereira et al. (2020a, b), and the foundations of which have been welcomed by the IPBES Plenary at its ninth session (IPBES 2022a).

The Nature Futures Framework focuses on the multiple types of values that underpin relationships between people and nature. It was specifically designed to bridge diverse ways that humans value nature in the efforts to create more nature-centred visions and scenarios. As there are many ways of ‘living in harmony with nature’, depending on what particular value perspectives on nature are considered to manifest ‘harmony’, the Nature Futures Framework builds on stakeholder consultations that generated a wide range of visions of desirable futures for biodiversity and people (Lundquist et al. 2017; Pereira et al. 2020a, b), as well as on the terminology used in the IPBES guidance on values that identifies intrinsic, instrumental, and relational nature values (Pascual et al. 2017; IPBES 2022b). The Nature Futures Framework places values that people have for nature at its core (IPBES 2022c). This focus differs from other global scenarios that have considered nature and people’s connection to nature as outcomes (Rosa et al. 2017).

**Fig. 1:**
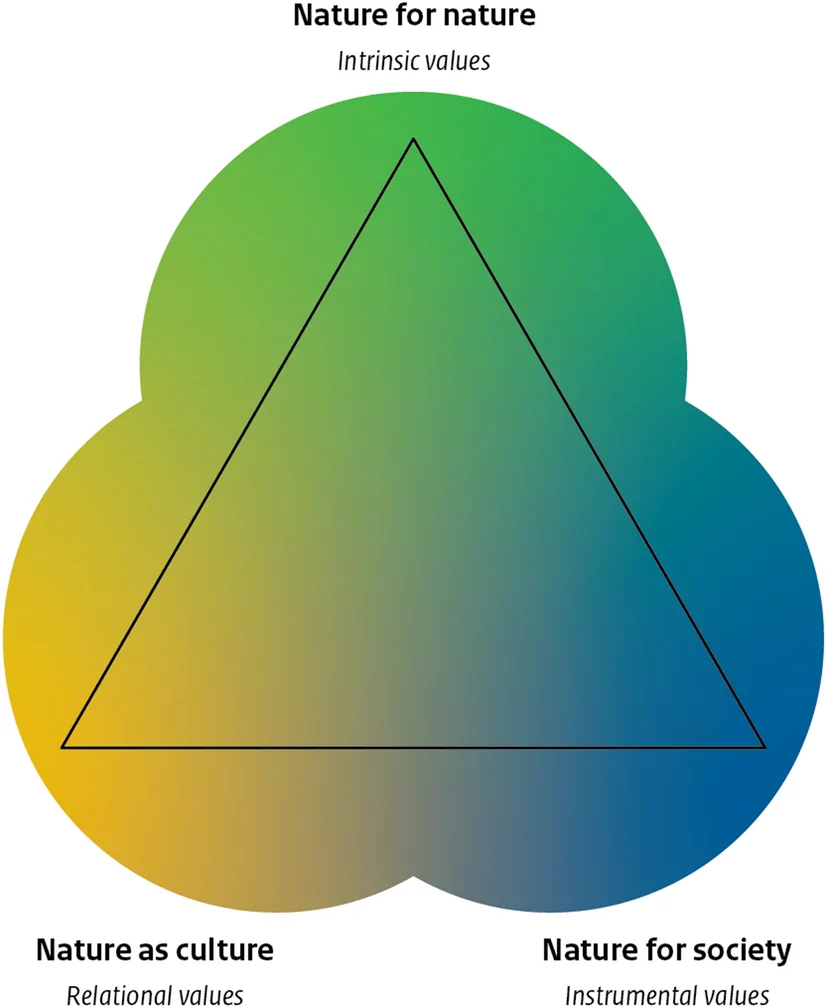
The Nature Futures Framework (NFF) and the three main value perspectives constituting the relative space within the NFF (from Pereira et al. 2020a, b; IPBES 2022a, b, c)

These diverse ways in which people value nature can be used to characterise a diverse range of relationships that people have with nature, and based on these, to develop possible future scenarios. The Nature Futures Framework identifies a minimal triangular space that represents the relative influence of three value perspectives on the relationship between people and nature: *Nature for Nature (NN)*, *Nature for Society (NS) and Nature as Culture/One with Nature (NC)* (Fig. 1). Relationships between people and nature can be visualised using this triangular space. Each corner illustrates a different type of relationship between people and nature—underpinned by its corresponding nature value—while the interior of the triangle represents a combination of these idealised types (Fig. 1), for example, reindeer pastoralism that is part cultural and partly for subsistence. Desirable futures for nature are represented within the triangle where nature is highly valued, whereas undesirable states for nature and people are represented by the space outside the triangle. Nevertheless, the framework does not aim to prescribe any particular narratives or scenarios as preferred based on their location in the Nature Futures Framework, reflecting that value preferences vary culturally and geographically (IPBES 2022a, b, c). The coloured circles (Fig. 1) associated with each value perspective blend together where they intersect, showcasing that they are not mutually exclusive (IPBES 2022a).

The Nature Futures Framework can be used to assess models and scenarios in terms of what types of nature values they emphasise (e.g. Quintero-Uribe et al. 2022). It provides a tractable way of organising multiple types of nature values, which allows the Nature Futures Framework to be applied across diverse social, geographical, and sectoral contexts. This pluralism is especially important in developing scenarios, because different values of nature continually co-exist, conflict, and combine and result in diverse configurations of human-nature relationships (Jacobs et al. 2020; Pascual et al. 2021). Currently, the IPBES task force on scenarios and models is developing methodological guidance to support the operationalisation of this framework by scientific communities and other stakeholders to use it to improve models and develop scenarios (IPBES 2022c).

In this paper, members of the IPBES task force on scenarios and models use the Nature Futures Framework to develop a methodological approach for the creation of narratives that characterise different types of people–nature relationships. The information captured in these types of narratives can be used to assess and develop scenarios and models whilst acknowledging the underpinning value perspectives on nature. We also present the narratives resulting from this methodological exercise, which can be used as illustrative examples for future work on the framework implementation and scenario development.

Narratives provide a frame in which to discuss and interpret quantitative results, as well as to highlight issues to model, and as a result they play a key part in the design of scenarios and models (O’Neill et al. 2017). The narratives developed here, referred to as illustrative narratives, seek to help users of the Nature Futures Framework to translate nature values represented within the framework’s triangle into concrete forms of people–nature relationships, such as the way we farm, acquire energy for living, or manage land for nature conservation. The term *illustrative* reflects that these narratives exemplify the multiple ways in which desired nature futures can be represented. The pluralistic and qualitative information captured by the illustrative narratives can be used as the basis for scenarios and models development. A prior step to narrative development is the characterisation of “scenario skeletons” (Alcamo and Henrichs 2008; Pereira et al. 2017), which represent a scenario’s main structure and core features. Scenario skeletons provide enough detail to communicate, compare, and elaborate the conceptual and technical components of a set of scenarios and models prior to their quantitative data analysis. If these skeletons are developed as full scenarios, it is then possible to link the latter to their corresponding underlying nature values.

This paper outlines the methods used to develop a set of scenario skeletons consistent with the Nature Futures Framework and its relative space, which were then used to build their corresponding illustrative narratives. We articulate lessons from this process that can be used to support the operationalisation of the Nature Futures Framework. The paper concludes by explaining how this work can contribute to the advancement of modelling multiple value perspectives on nature, and provide some suggestions on how to further operationalise the Nature Futures Framework to facilitate scenario development.

## Materials and methods

In February 2020, the IPBES task force on scenarios and models organised a workshop in Shonan Village, Hayama, Japan, to develop illustrative scenario narratives from the Nature Futures Framework (PBL 2020). Nine members of the task force attended in person and 24 remotely due to the COVID-19 pandemic. There were also follow-up online activities among the task force to elaborate, revise, and refine the developed narratives and methodology (PBL 2020).

The goal of the workshop was to test the Nature Futures Framework by developing a set of scenario skeletons for “new narratives for nature”, using insights gained during previous consultations and workshops (Lundquist et al. 2017; PBL 2019a, b). This was done through four steps described below: (1) reviewing the Nature Futures Framework and its triangular space; (2) defining scenario skeletons through thematic characterisation; (3) developing illustrative narratives within the Nature Futures Framework space; and (4) aligning and comparing narratives.

### Reviewing the Nature Futures Framework and its ‘relative space’

The Nature Futures Framework illustrates how it is possible to acknowledge a diverse mixture of values of nature depending where in the triangle one is situated. Accordingly, different locations within the triangle are associated with different combinations of specific nature values, represented in each of the corners of the triangle: intrinsic, instrumental, and relational. In this regard, the triangle of the Nature Futures Framework can be understood as a ‘relative space’. The mixture of nature values associated with a location has to be coherent and consistent with the surrounding location’s values, including the three main corners. For instance, if one is situated at the ‘Nature for Nature’ corner where the intrinsic value is at its maximum and moves halfway towards the ‘Nature for Society’ corner (Fig. 1), the nature values and its corresponding people–nature relationships represented in this new location must coherently and consistently incorporate elements of instrumental values represented by the ‘Nature for Society’ corner.

**Fig. 2:**
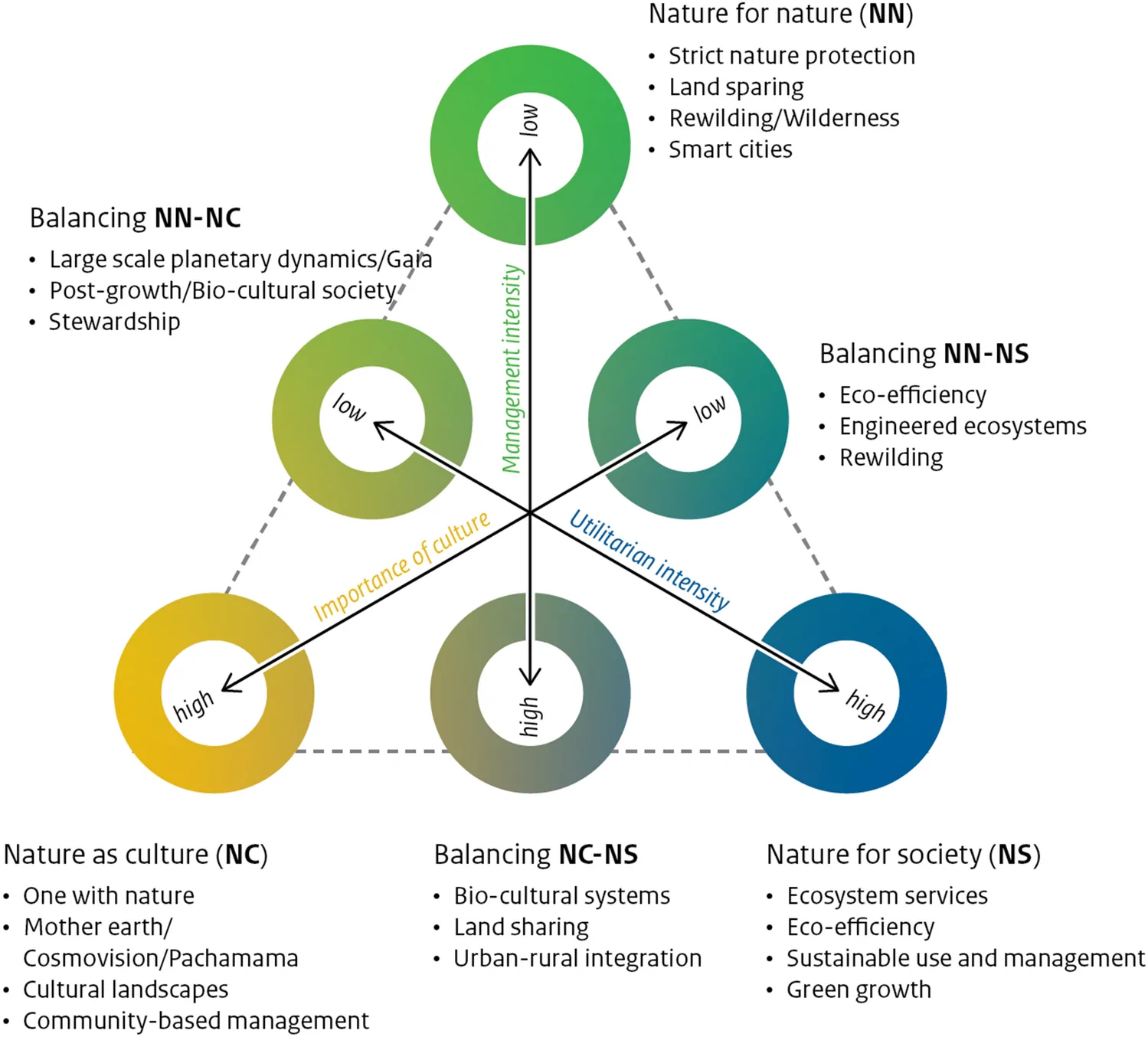
Value perspective locations (circles) and descriptive characteristics (bullets) of the illustrative narratives. These scenario skeletons lay within a fully relational space (axes); the position of each scenario skeleton is dictated by its relation to the adjacent and opposite one. Adapted from Kim et al. (2021)

Exploring the relativity of the triangle space was the first step of the process of narrative development, whereby we identified the locations within the triangle to be developed into scenario skeletons, whilst outlining the mixture of interconnected nature values associated with these locations. To this end, workshop participants chose six locations within the Nature Futures Framework (Fig. 2): the three corners of the triangular space (extreme value perspectives) and the three sides (locations that represent a combination of two corresponding value perspectives). A seventh location was considered, in the centre of the triangle space—consisting of a mix of the three value perspectives in the corners of the triangle, but we decided not to characterise it because the extremes and combinations of two values were clearer and allowed for paired comparisons ("Aligning narratives to capture the nature values gradient").

A preliminary characterisation of the nature values was done by reviewing and extracting information from IPBES workshops’ reports (Lundquist et al. 2017; PBL 2018, 2019a, b; Pereira et al. 2020a, b), and by considering, in particular, the visions of desired nature futures that were created during a large stakeholder workshop in Auckland, New Zealand, in 2017 (Lundquist et al. 2017). The seven visions resulting from the Auckland workshop emphasised different preferences for people–human relationships and could be, therefore, distributed across the triangle space based on their associated underlying nature values (PBL 2019a). For example, while some visions emphasised the indirect and intangible benefits of biodiversity, others emphasised the direct uses of nature (Lundquist et al. 2017). We note that, while these visions were considered a logical starting point for the sense-making of the locations in the triangle, the framework offers enough flexibility to implement other participatory approaches to identify locations within the triangle and characterise their nature values.

In the process of defining the interdependence among locations and corresponding nature values, it was important to identify common features that could be considered desirable across the futures. For instance, futures where the Sustainable Development Goals (UN 2016) were met was considered as a starting point, recognising that there are several possible pathways to meet these goals and that each would result in a different future. For example, we included poverty eradication (SDG 1), nutritious diets (SDG 2), access to clean water (SDG 6), and gender equity (SDG 5), among others. In addition, it was considered necessary for the direct drivers of climate change to be managed for the futures to be ‘desirable’. This includes futures where strong climate mitigation has limited global warming to 1.5–2 °C, habitat loss and overexploitation have been halted, and pollution has been greatly reduced.

### Defining scenario skeletons through thematic characterisation

The six identified locations across the triangle of the Nature Futures Framework provided a relative space of nature values that permitted the characterisation of each scenario skeleton, which in turn would establish the structure of the illustrative narratives. At this point, it was important to perform a structured characterisation of scenario skeletons, since this would allow us to systematically compare the resulting narratives to extract their differences and similarities (further details in "Developing illustrative narratives within the Nature Futures Framework space" and "Aligning narratives to capture the nature values gradient").

To build each skeleton, we characterised a set of themes which were expected to be important components of social–ecological systems (e.g. trade, agriculture, law, energy) and should therefore be addressed in scenarios that describe desirable nature futures (Table 1). Categories of the STEEP framework (Social, Technological, Economical, Environmental, Political), which is widely used in strategic foresight (Schultz 2015), were used as a starting point to elaborate a total of 22 scenario themes. The thematic characterisation for each skeleton was performed using a cross-comparison table, where columns represented skeletons and rows represented the themes. Using this table, participants discussed how the six scenario skeletons differed from one another in terms of structure, functions, feedbacks, and how changes from the current world could produce these future visions. This imaginative exercise was designed to push beyond the boundaries of existing frameworks to ensure that six distinct desirable futures would capture diverse manifestations of people–nature relationships. The theme characterisation focused on one theme at a time (i.e. row by row) and considered the relativity of each theme across the six skeletons. At the same time, participants checked the coherence and consistency within each scenario skeleton across the themes. After describing the themes with the use of a cross-comparison table, participants titled the scenario skeletons with names that emphasised their corresponding value perspectives. The full cross-comparison table is presented in the supplementary information (ESM 1).

**Table 1:** Twenty-two scenario themes that were organised into five overarching grouping categories for scenario description (further details in "Defining scenario skeletons through thematic characterisation" and "Developing illustrative narratives within the Nature Futures Framework space")

Society’s governance	Society’s functioning	Natural resource management	Habitat and biodiversity	Society’s organisation
Economy	Infrastructure	Food	Megafauna	Trade
Governance	Energy	Diet	Oceans	Law—rights
Cities	Transport	Agriculture	Biodiversity use	Education
Communities	Water	Fisheries		Policy
		Aquaculture		
		Land management		
		Well-being		

### Developing illustrative narratives within the Nature Futures Framework space

The scenario themes were grouped into five overarching categories to provide a coherent structure for writing the illustrative narratives (Table 1). These categories were designed to group closely related themes so that similar themes were considered together in narrative development to promote consistency and efficiency. Participants wrote narratives for each scenario skeleton following a standard format based on one paragraph per overarching category. Drafting the paragraphs used the information in the cross-comparison table from Step 2.2 to ensure that each of the 22 themes were addressed within the five overarching categories. This standardised format for the six narratives enabled them to be systematically compared. Based on the captured similarities and differences, we then distributed narratives throughout the relative space of the triangle (see details in "Aligning narratives to capture the nature values gradient").

### Aligning narratives to capture the nature values gradient

A key objective of illustrative narratives is to acknowledge and track the (often implicit) underlying nature values of scenarios and models. To this end, it is important to be able to evaluate how the different elements of each narrative are interlinked, and how these would be differentiated and quantified in subsequent scenarios and models. To explore this, we arranged the six skeletons along three gradients represented by each of the three areas of the transformative change debate, specifically, the land sharing versus land sparing debate (Loconto et al. 2020), the Half Earth or Whole Earth approach (Büscher et al. 2017), and the green growth versus post-growth economic paradigms (Otero et al. 2020). These three areas were chosen to represent relevant debates that can be explored and analysed using scenarios and models. Each of the three gradients has two opposite points of view represented in the debate, with associated value perspectives on nature. We positioned the six skeletons along these three *nature value gradients* whilst maintaining a coherent interdependence of their characterised themes and nature values*.* Specifically, this exercise followed Hegel's system of dialectics in logic, where the “opposing sides” are different definitions of logical concepts that are opposed to one another (Hegel 2014). We considered this to be a useful framing, because it does not mean that one side is ‘right’ while the other is ‘wrong’, but, instead, says that they are opposing logics that cannot be simultaneously maintained, i.e. it is not possible to simultaneously have land sharing and land sparing, but it is possible to hold a relative space between them. Further, this is by no means an exhaustive list of transformative change interventions, but it allowed the skeletons to illustrate how diverse concepts on transformative change could be included in future scenarios for nature, whilst maintaining internal coherence.

To capture the relative positions of narratives along the gradients, we performed a paired comparison among narratives to extract and summarise specific differences and similarities. This paired comparison was facilitated by the structured format of the narratives, which followed the five overarching thematic categories. The full table with the paired comparisons is presented in the supplementary information (ESM 3).

## Results

### Relative space within the Nature Futures Framework and scenario skeletons

Nature values of six locations within the triangular space are reflected in the characterisation of the scenario skeletons (Table 2). Since the three corners of the triangle represent the extreme value perspectives, their corresponding scenario skeletons were dominated by such a value perspective. The corner position of the triangle where the ‘Nature for Nature’ (NN) perspective is located is reflected by a scenario where nature’s value is intrinsic. This skeleton envisions a future where wilderness is dominant and therefore management intensity is low (Fig. 2). To characterise a society that functions under an intrinsic value for nature, participants described themes that pushed the boundaries of technology, architecture, governance and land management so humanity would minimise its scope while maximising nature’s. Accordingly, this skeleton was named ‘Arcology’ (NN, Table 2). The scenario skeleton that sits directly opposite the NN corner of the triangle is positioned between the ‘Nature as Culture’ (NC) and ‘Nature for Society’ (NS) corners, and therefore reflects a balanced point between the relational and instrumental nature values of these two corner perspectives (i.e. NC–NS; Fig. 2). The NC–NS location also reflects values that are coherently oppositional to the NN corner. As such, although this scenario skeleton envisions a context where management intensity is high (Fig. 2), the use of nature is innovative and respectful, therefore capturing the desirable relational and instrumental values represented in its location in the triangle. The resulting skeleton from this location was named ‘Sharing through sparing’ (NN–NS, Table 2).

**Table 2:** Overview of scenario skeletons and associated nature value perspectives

Title	Value persp.	Two-line summary	Key words^a^
Arcology	Nature for Nature (NN)	People respect and value all life on Earth intrinsically. This world is characterised by extreme land sparing, as vast areas of land and sea are strictly protected. People live in dense self-sustaining urban areas designed to minimise the influence of people in the biosphere	Planetary stewardship, post-growth, smart cities, blue-green infrastructure, protected area, large-scale ecological dynamics, rewilding, self-sufficient settlements
Sharing through Sparing	Balancing Nature for Nature and Nature for Society (NN–NS)	People have a fairly strong use orientation towards nature, but also value and protect the self-regulating capacity of the biosphere as biodiversity and natural processes provide the resilience that enables humanity to stay within planetary boundaries. While sparing space for nature, remaining areas are used intensively, but efficiently and sustainably	Eco-efficiency, green growth, blue-green infrastructure, urban–rural integration, optimised ecosystem services, protected area, engineered ecosystems, rewilding
Optimising Nature	Nature for Society (NS)	A highly connected world that shares knowledge and technology to maximise efficient and sustainable utilisation of nature’s contributions to people while ensuring maintenance of the key ecosystem functions that support them	Eco-efficiency, green growth, smart cities, urban–rural integration, land sharing, optimised ecosystem services, engineered ecosystems
Innovative Commons	Balancing Nature for Society and Nature as Culture (NC–NS)	People have built a world of innovative ecological commons and live in interconnected blue-green cities and rural settlements across land- and seascapes. People use their local and traditional knowledge, and technology, to manage and expand the use of ecosystems and biodiversity also to enhance their culture	Bio-cultural heritage, commons, post-growth, blue-green infrastructure, urban–rural integration, cultural landscapes, land sharing, optimised ecosystem services
Reciprocal Stewardship	Nature as Culture (NC)	In this world, values of reciprocity and harmony structure peoples’ relationships with nature at all levels of human organisation. Biological and cultural diversity are co-conserved and co-managed across a wide range of interconnected bio-cultural systems	Bio-cultural heritage, stewardship, commons, post-growth, cultural landscapes, engineered ecosystems, self-sufficient settlements
Dynamic Natures	Balancing Nature for Nature and Nature as Culture (NN–NC)	Dynamic, connected and biodiverse ecosystems are valued to allow traditional socio-cultural reproduction, spiritual values and connections to be re-established and new ones to be shaped. Society accommodates the dynamism of nature through both traditional and innovative lifestyles that takes into consideration cultural heritage and traditional ecological knowledge	Planetary stewardship, post-growth, urban–rural integration, engineered ecosystems, large-scale ecological dynamics, rewilding, self-sufficient settlements

^a^See Annex I of IPBES (2019) for a glossary that defines many of these key words

The scenario skeleton located in the ‘Nature for Society’ corner emphasises an instrumental value perspective for people–nature relationships. While still sustainable, this skeleton frames a society that seeks to optimise the use of nature for people’s benefit, thus it is named ‘Optimising Nature’ (NS). In the opposite location, between ‘Nature for Nature’ (NN) and ‘Nature as Culture’ (NC), instrumental values towards nature are low (Fig. 2). Here, the scenario skeleton envisions a future where nature is lived as a culture that responds to the needs of people and communities while balancing the intrinsic values of nature. This skeleton was named ‘Innovative Commons’ (NC–NS).

The scenario skeleton located in the ‘Nature as Culture/One with Nature’ corner reflects a future with high relational values (Fig. 2), where values of reciprocity and harmony drive people’s relationships with nature at all levels of human organisation. This skeleton was named ‘Reciprocal stewardship’ (NC). The scenario skeleton opposite this corner, between ‘Nature for Nature’ (NN) and ‘Nature for Society’ (NS), reflects a vision with low relational values (Fig. 2). Here, the scenario skeleton envisions a society with a fairly strong use orientation towards nature, but that recognises that broad extents of wilderness are required to allow for the fundamental processes of nature. This skeleton was named ‘Dynamic Natures’ (NN–NC). Table 2 presents an overview of the comparison table and a summary of each of the scenario skeletons.

### Illustrative narratives

Based on the characterised themes and skeletons, participants developed six narratives that followed the same structure. Because certain themes were more central to some scenario skeletons than others, the extent to which each specific theme is addressed varied. Below, we present a summary of each narrative (approximately 600 words), and a two-line summary of each narrative can be found in Table 2. Full narratives (1,000–1,500 words) are in ESM2.

#### Narrative: Arcology (Nature for Nature)

This world is built on the *Nature for Nature* perspective. In this vision, people respect and value all life on Earth because it has intrinsic value. The world is characterised by extreme land sparing with optimal use of space within cities, while vast areas of land and sea are strictly protected. This alludes to the spirit of the Arcology—or spaceship living—built on high efficiency, no waste principles, and strongly regulated behaviour. People live in dense self-sustaining urban areas designed to minimise the impact of people in the biosphere and to preserve wild, autonomous nature.

To ensure highly connected wilderness areas and effectively preserve wilderness, more than 70% of natural areas are strictly protected. The high seas are designated as Marine Protected Areas with no take zones. All people live in high-tech cities that are very efficient in water use and recycling and designed to minimise pollution and the impact of resource extraction. In these city-states, the economy is mostly based on services and knowledge production. The city-states are controlled and operated under a strong and effective governance at the global level facilitated by highly structured international cooperation.

Human infrastructures are exclusively limited to urban areas and optimised to be respectful to the environment whilst adequately meeting the needs of the population. Underground hyperloops and drones are used to connect cities to minimise anthropogenic impacts on nature. In the interests of efficiency, the production of energy is highly optimised at large scale to supply the city-states and especially the data centres. All the energy is produced by the new generation of thermonuclear fusion reactors for a clean energy transition. To preserve water resources, the natural water cycle runs with little human intervention and all internal city water is cycled with the highest efficiency.

The management of microbiological processes is implemented for the delivery of nature’s contribution to people within cities (e.g. microbiotic systems for sanitising water quality, food production, etc.). To meet nutritional outcomes, technological advancements such as laboratory-grown meat have optimised meals to improve flavour and mimic cultural diversity of foods. Fresh food is limited to vertical urban gardens. The catches from marine and freshwater fisheries are limited, as most ecosystems are set aside as protected areas. Aquaculture production is restricted to specific areas and dominated by seaweed at scales that are well within ecological capacity and make use of technological processes like microalgae production.

Well-being is primarily generated through virtual reality and supported by smart technological systems based on the Internet of things. People willingly accept restrictions in their occupation of space. Environmental conservation and protection are the highest priority in constitutional law and by-laws. Therefore, environmental principles are integrated in all components of education. Policies enforce conservation and environmental laws and aim at facilitating their translation into the lives of people in city-states. Global policies and regulations establish political and diplomatic relations as well as norms of communication and behaviour outside the megacities and in the ways the arcologies must exchange and live with each other. To this end, although there is a high efficiency of material recycling, mining takes place under strict global protocols and can only take place underground, with no impacts to be felt in the biosphere. Asteroid mining is being contemplated to address these concerns. Urban security has precedence over personal privacy and this is reflected in preeminent policies, regulations, and laws which are carefully monitored and enforced.

#### Narrative: Sharing through Sparing (Nature for Nature/Nature for Society)

This vision sits in between *Nature for Nature* and *Nature for Society*. Societies have a fairly strong use orientation towards nature. However, people recognise that biodiversity and natural processes are fundamental to the resilience of the biosphere and enable humanity to stay within planetary boundaries. Thus, people do not seek to fully control, engineer or optimise the natural world. Rather, there is a deeply rooted understanding that benefitting from nature’s services, especially regulating services on a global scale, requires allocating and protecting extensive areas on the planet where natural dynamics can occur at large scale and biodiversity can thrive, aligning with a ‘Half Earth’ vision. Remaining areas are used intensively, but efficiently and sustainably.

Protected areas (PAs) are the primary tool to enable wilderness and natural dynamics. PAs focus on those areas that matter most for safeguarding the self-regulating capacity of the biosphere. No predetermined percentage of PAs is pursued; the percentage results from an evidence-based assessment of earth system science combined with algorithmic optimisation that considers human rights. An accounting system is in place to distribute costs and benefits related to the protection of nature among nation states and their citizens, supported by a mediation and arbitration institute that resolves conflicts. Extraction of natural resources outside PAs is heavily monitored and controlled to achieve sustainability, for which international treaties are in place. For marine systems this results in very limited bycatch, with all destructive fishing methods prohibited and historically collapsed stocks rebuilt. In terrestrial areas, where society does not let nature run its course, people engineer with nature to optimise ecosystem services.

Cities are nature inclusive and redesigned to cope with sustainability challenges and natural disasters. Urban lifestyles are fairly homogeneous and resource efficient, as an important part of people's lives unfold online. Tensions in communities that may result from this lifestyle are prevented through social innovation, with a large role for education. A combination of highly engineered nutrition-balanced diets with a range of fresh local and seasonal produce contributes to healthy lives. Governance is decentralised to a scale determined by urban areas and their surrounding landscapes, seascapes and protected areas. Outdoor agriculture generates high yields by making use of ecological principles and is complemented with high-tech greenhouse horticulture minimising resource input and maximising recycling to prevent pollution. This includes vertical horticulture in cities such as hydroponics and aeroponics. Aquaculture occurs in designated areas where nutrients are circulated as much as possible and focuses on low trophic level species (e.g. algae, bivalves) and multi-trophic systems of ‘high productivity, high protein' species. International trade is moderate, enabling geographically optimised generation of provisioning services.

Cities rely on local and regional energy generation from an optimal mix of renewables with smart grids. All water-, heat- and energy-use systems are within a circular economy framework with local and regional production, management and use. Transport is clean, efficient, and fossil fuel free, with dominant use of public transport for short to medium trips and limited use of personal transportation. Global and long-distance transportation is largely by air (clean blimp tech) and hyperloops. The economy has shifted to a green, interconnected market economy that is decoupled from environmental impacts, including telecoupled ones. Environmental sustainability is thus prioritised over economic growth. The private sector operates within a strict framework of rules, regulation, taxes, and natural capital accounting. Land-use and tenure regulations allow for productive use, with some protectionism limiting potential negative impacts on biodiversity and climate. Policies emphasise the primacy of global security over privacy rights with strict measures on protected areas and prioritising environmental and sustainable economy policies.

#### Narrative: Optimising Nature (Nature for Society)

This vision is based on the *Nature for Society* perspective. Societies seek to maximise efficient and sustainable utilisation of nature’s contributions to people, while ensuring maintenance of the key ecosystem functions that underlie them. The world is highly organised and regulated through top-down, centralised governance systems with a high degree of global cooperation. Following an emphasis on green growth, governmental institutions work closely with the private sector to advocate evidence-based decision-making that ensures resources are used efficiently and distributed equitably. Technological innovations are co-developed between producers, researchers, and industry to make use of local biodiversity, and to assess and monitor optimal ways of utilising nature’s contributions to people over time. Most people live in high-tech cities that are designed to maximise the efficiency of resource use and to support the sustainable delivery of multiple urban services, for example through pollination corridors, vegetated buildings, and artificial wetlands. Cities are highly connected through the transfer, sharing and trading of goods, services, knowledge, and technology. Cities are also highly connected with surrounding clusters of rural settlements.

Societies appreciate “tamed” nature which stimulates innovative entrepreneurship across urban–rural landscapes and localised ecosystem services flows (e.g. closing the nitrogen cycle at the landscape scale). Development projects employ natural capital accounting and focus on nature-based solutions for securing long-term prosperity. The ecologically literate population has a high awareness of the consequences of lifestyle choices, and the role of women’s knowledge in the sustainable use of biodiversity is well recognised and valued. People use environmentally friendly carbon-neutral public transport for connectivity across the planet. This reduces access inequalities and enhances liveability of rural areas. Energy supply is from renewable energy sources: decentralised, but connected by efficient ‘smart’ energy grids.

Food systems are global and fully integrated. Large international corporations together with effective government regulations ensure that production systems are highly efficient with low ecological impacts. Food processing technology is advanced, resulting in almost 100% use of biomass and nutrients from food products. Land use is multifunctional, and managed sustainably within a landscape matrix that supports ecosystem functioning, whilst delivering multiple co-benefits of NCP (Nature’s Contributions to People) and not simply food. This includes agro- and mixed-forestry systems, wetlands, and connected habitat mosaics to provide recreation and aesthetic value as well as supporting ecosystem resilience. Large land areas are used for crop and livestock production due to agricultural extensification, but to minimise trade-offs with nature, practices are biodiversity friendly, avoiding excess nutrients from fertilisation and minimising waste. Deforestation is avoided through restoration of degraded lands for production. Genetically modified crops are socially accepted and widely used. Almost all aquatic systems are used for food production from fisheries and aquaculture including the high seas and Exclusive Economic Zones, but technology allows for precise extraction of biomass to maximise for ecosystem-level production with low biodiversity loss. Incentive systems allow for efficient and effective control, monitoring, and regulation. Ecological extensification happens in aquaculture in both freshwater and marine systems with new multi-trophic aquaculture techniques and systems allowing for efficient utilisation of nutrients with minimum ecological impacts.

New knowledge and technology allow for precise and effective allocation of water resources to maximise its benefits to people, whilst ensuring environmental flows to support aquatic ecosystems. Other non-food extractive uses of natural resources, such as energy production and mining, take place on land and at sea. The use of data, technology, and strong multi-level governance ensure accurate environmental impact assessments. Limited losses of biodiversity and landscape modifications are considered socially acceptable if they do not adversely affect the long-term delivery of nature’s contributions to people. The few protected areas that exist safeguard key ecological functions that are essential for supporting nature’s contribution to people.

#### Narrative: Innovative Commons (Nature for Society/Nature as Culture)

This world sits between *Nature for Society* and *Nature as Culture*. In this vision, people have built a world of innovative ecological commons and live in interconnected blue-green cities and rural settlements across landscapes. Nature is lived as a culture that responds to the needs of people and communities, recognising a land sharing perspective where most biodiversity is conserved through use. This is made possible by a thriving blue-green social economy (i.e. reliant on circular principles and local solutions) that is interconnected through equitable trade of goods. The economy is regenerative: it does not just use markets, but creates new economic value through diverse value streams (e.g. domestic labour and community work), breaking away from profit-maximising exploitative relations. A wide diversity of socially oriented organisations (e.g. cooperatives, associations, social enterprises, community-based and integrated landscape initiatives) and institutions give shape to the social nature of the economy.

Governance is largely decentralised, but emphasises links between urban nodes to well connected rural groups across regions. Global governance is based on equitable representation of strong, autonomous regions. It strengthens a global movement for regional, decentralised systems that ensure that benefits do not accrue only to powerful actors. Medium-sized blue-green cities are designed around community-friendly ecological principles to deliver services, from spiritual gardens to universal clean water. They link with rural settlements to form a network of interconnected semi-autonomous entities across the landscape. Advanced nature-based technologies are shared and balanced by the decentralised nature of the digital commons and by P2P (peer to peer) networks that link virtual communities with “real” rural and urban communities of practice. A strong place-based cultural identity is diffused and shared throughout community-based networks and collaborative commons.

Transport is multimodal, using public as well as individual means of transport by land, air, sea and river, but it is based on innovative, low-impact technologies that connect people and goods locally and regionally. The energy system is a collaborative energy regime in which millions of people produce their own renewable energy (solar, wind, thermal, etc.) with the help of medium and micro–power plants, as well as advanced storage technology, including hydrogen, to store intermittent energy. Excess energy is freely traded over the energy Internet by autonomous energy-producing and -consuming communities. Commoning, reciprocal credit, and barter trade are essential and prevail over old supply and demand market pricing.

The overall ecological infrastructure is based on a combination of traditional practices and novel technologies and targets the conservation of culturally significant species in community conserved areas and co-managed landscapes. Protected areas represent no more than 14% of land areas, mainly in the form of community conserved areas and used mainly for eco-tourism. In marine systems, people practise ecological restoration and management of marine resources to sustain local economies and maritime cultures and trade. Communities use their local and traditional knowledge, and technology, to expand the use of biodiversity and also to enhance understanding and recognition for various cultures among communities. Natural biological resources (e.g. nutraceuticals) are widely accessed and sustainably used. Benefits arising from genetic resources are equitably shared.

An important part of policy is to secure free access to a 10G-1Q worldwide web through advanced types of open-source licences. Policies incentivise knowledge–policy integrations that facilitate resource-saving innovations and allow for more ecological production. Formal regulations are limited by the primacy of citizen networks and informal agreements and a strong role of education systems. Networked citizens form a strong basis for environmental awareness and are actively engaged with political processes and law enforcement. Laws emphasise community rights, and citizen participation through economic cooperation in the commons is incentivised.

#### Narrative: Reciprocal Stewardship (Nature as Culture/One with Nature)

This vision illustrates a world where values of reciprocity and harmony drive people’ relationships with nature at all levels of human organisation. It sits at the *Nature as Culture* corner of the Nature Futures Framework. Biological and cultural diversity are co-conserved and co-managed across a wide range of interconnected bio-cultural systems.

This vision is supported through governance processes that take precedence at the scale of self-determined jurisdictions, rather than nation states, resulting in a rich diversity of governance systems. The latter recognise Indigenous people’s sovereignty over their lands and knowledge systems, and capture the identity and needs of local communities. Restricting the access of people to nature’s benefits is anathema in this future, echoing a Whole Earth approach. The wide variety of governance systems is challenging to manage in an integrated way, since these are very context dependent and self-determined. Nevertheless, the shared fundamental values towards nature facilitate cross-system interactions. Horizontal governance systems work at the level of small cities, interconnected with a patchwork of autonomous rural settlements.

Globally, the world is post-growth. Economic exchange focuses on the social value of things rather than their monetary value. The development of new metrics such as a new Gross National Happiness Index are vital to guide regional and international collaborations. Technology is advanced, but it is specialised for functions that reinforce interpersonal relations and cultural connectivity with and through nature. Communities live connected to nature through evolved and traditional practices co-developed with the latest technology, thus creating a resilient and functional continuous bio-cultural landscape.

Infrastructure is designed to handle small-scale processes, activities and community needs. Transport is based on multimodal travel (e.g. bikes, horses, shared cars). This infrastructure enables trade of local products within regions. Infrastructure for energy is decentralised: each building and system produces its own energy from different renewable sources of energy. The design of freshwater infrastructures stems from the rooted socio-cultural value towards this “living system”.

Food production is small scale and for local consumption, based on the cropping and harvesting of a wide diversity of edible species. Food consumption patterns are highly seasonal, and the cultural significance of eating is a core value. The maintenance of indigenous and traditional practices is fundamental within production systems. Traditional aquaculture (e.g. clam gardening, mixed agriculture–fish pond) complements agriculture, as does the harvesting of crops’ wild relatives, resulting in highly heterogeneous land and seascapes. These types of productivity systems are only attained with strong collaboration within families and communities, which is fostered by a strong sense of place and spiritual connection with nature, all contributing to health and well-being. The strong sense of place, cultural identity, and mental and spiritual connection with nature, all contribute to a good quality of life.

The persistence of terrestrial and marine biodiversity and associated ecological processes are secured through traditional stewardship. Culturally significant species are conserved at the expense of others in co-managed land- or seascapes with no protected areas that keep people away from the land. Deep relational values with nature have established a fundamental understanding about the complexity of ecosystems and permit practical, integrated conservation of land/seascapes and species. Respectful dialogue between indigenous and local knowledge systems and science facilitates community resource management and maintains cultural identity through sustainable consumption of wild species.

Laws emphasise community rights, socialisation is high, and enforcement is done mainly through social networks, and in conformity with social norms. Policies and regulations aim at reinforcing the cultural fusion of ecological dynamics with community histories and priorities. Much of this effort is directed towards the educational system and social institutions, and largely governs reciprocity between people who use the abundant harvests of nature and give back by nurturing nature.

#### Narrative: Dynamic Natures (Nature as Culture/Nature for Nature)

This narrative sits between *Nature for Nature* and *Nature as Culture*. Human societies respect, value, and accommodate the dynamism of nature through both traditional and innovative lifestyles that take into consideration the natural systems' resilience, cultural heritage and traditional ecological knowledge. Healthy and biodiverse ecosystems enable traditional socio-cultural reproduction, spiritual values and connections to be re-established and new ones to be shaped.

This is a polycentric world of nested social–ecological systems governed by largely self-sufficient units that are defined by their ecosystem rather than social boundaries (e.g. watersheds, biomes). Nations states are no longer necessary and the world has moved beyond measuring development through growth metrics, reflecting a post-growth society. Whilst there is limited global trade and resource sharing, there is a high level of cooperation between the units to ensure global compliance with global environmental legislation for the freedom of movement of all species and for regional knowledge sharing and governance. Adaptive and dynamic land and water management practices account for the season and geography, and are very context dependent and flexible.

Water is identified as key to life resulting in a strong demand for the recognition and restoration of the socio-cultural role of rivers as living systems. These now have legal standing, together with the environment as a whole. Rivers flow freely without impediment. People use local and less globalised resources within circular economies, and focus on the creation of dynamic ecological infrastructure with much fewer roads. This means there are no more dams or large-scale permanent inorganic infrastructure.

Human settlements are dynamic and adapted to the movements of nature while being flexible (some nomadic) to ecological shifts using innovative technologies such as floating houses. Adaptive, dynamic transport uses tides, wind power and new technology that is able to capture these natural forces, building on traditional knowledge (e.g. Polynesian/Pacific Islander boats). Travel by air and sea is therefore enhanced, making the planet truly connected. To consume the minimum amount of resources, buildings are well integrated with their environment (in some instances being embedded within hills (like hobbit homes) or cave dwellings). There is a community-driven demand for decentralised, local control over resources. Energy is produced from renewable sources. More effort is put into reducing energy consumption than producing it and so each community is energy secure and self-sufficient.

Diets are diverse and seasonal, based on what can be grown locally and ecologically without monocultures. Food production relies on harvesting from traditional production systems that have evolved with and are adapted to ecological dynamics. Pastoralism and gathering of wild fruits and cultivation of short-season crops are preferred over permanent agricultural structures. Agro-ecological landscapes, including agro-forestry, are linked with traditional technologies (e.g. terracing). Fishing is limited to traditional grounds within exclusive economic zones, dominated by small-scale fisheries, with limited production of high-value food products that are primarily to support local communities and livelihoods. Community-based and ecosystem-based fisheries management are in place. Traditional multi-trophic eco-aquaculture systems as well as aquaculture are tightly integrated with agricultural systems.

There are no protected areas per se, but rather entire ecological communities are protected in situ (land and sea) as this is especially important for the conservation of migratory species. Many species are indirectly conserved as people make way for nature and benefit from connected dynamic ecosystems, including novel dynamic conservation areas within a broader cultural landscape that recognises the rights of local communities. Ecological laws are enforced by community monitoring through citizen networks using the latest in drone and other non-invasive technologies. Strong environmental education is developed based on different cultural/traditional backgrounds and contexts. Every person feels connected to their community and values of reciprocity, harmony and relationality that reflect a variety of shifting relationships with nature.

### Capturing gradients in nature values

**Fig. 3:**
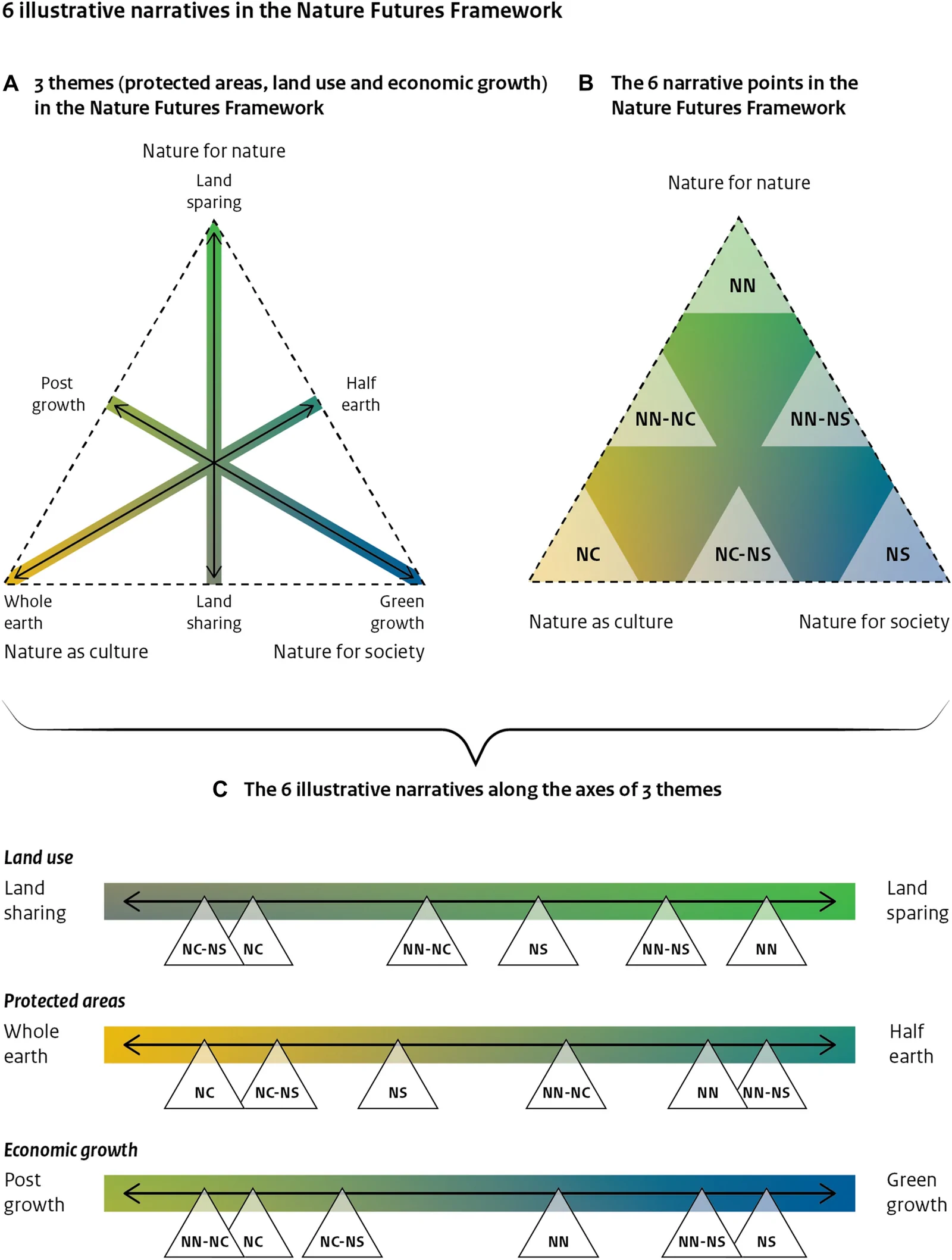
Summary descriptions of the illustrative narratives across three thematic groups, illustrating how the stories are diverse and contrasting, yet coherent and consistent

The similarities and differences between narratives extracted from the paired comparison allowed us to arrange the six skeletons along three gradients relevant to debates in transformative change, thus exploring interdependencies between the underlying values of each skeleton. With a fundamental characterisation of ‘Nature for Nature’ as leaving space for nature separate from humanity, *Arcology* (NN) sits at one extreme end of the land sharing, land sparing dialectic, with *Innovative Commons* (NC–NS) sitting at the opposite end, where land sharing is the most common practice, due to the ‘commons’ aspect in this narrative (Fig. 3c). *Dynamic Natures* (NN–NC) was seen to be most at the centre of these extremes, with *Reciprocal Stewardship* (NC) tending more to the land sharing side and *Sharing through Sparing* (NN–NS) lying closer to the land sparing extreme (as defined in its name).

The alignment of the narratives along the protected areas gradient, characterised by the Half Earth vs Whole Earth debate, is similar to the land-use gradient described above (second gradient in Fig. 3c). In this instance, *Reciprocal Stewardship* (NC) sits at the extreme end of that dialectic as it is defined by no separation between nature and society (i.e. the Whole Earth approach with no strict protected areas that divide people from nature) and so the opposite end of the gradient (i.e. the Half Earth approach with strict protected areas measures), is found within *Sharing through Sparing* (NN–NS; the side directly opposite the ‘Nature as Culture’ corner of the triangle). The other narratives then map onto this gradient in terms of the degree to which protected areas are relevant within the scenario narratives. Hence, *Optimising Nature* (NS; the ‘Nature for Society’ corner) was seen to lie between the two extremes of the gradient, with *Dynamic Natures* (NN–NC) further towards Half Earth, and *Innovative Commons* (NC–NS) slightly further towards Whole Earth. It was clear in the writing of the narratives that in terms of how land is allocated for use, *Arcology* (NN) and *Sharing through Sparing* (NN–NS) were closest in terms of how protected areas were deployed as a potentially transformative intervention. This relationship of land allocated for different uses was reinforced in both the first and second gradients, where you can see these two narratives cluster somewhat.

The final gradient is one of economic growth. It is plausible that the full gradient from green growth at one extreme to post-growth at the other could fit most of the narratives as they currently stand. However, this is an important debate around transformative change, so it was important to test how the narratives could map onto this gradient. The scenario most closely aligned with the green growth perspective is *Optimising Nature* (NS), at the ‘Nature for Society’ corner, reflecting the current dominant instrumental value perspective. This meant that the post-growth narrative would align with the opposite location to *Optimising Nature* (NS), that is, *Dynamic Natures (NN–NC)*. This was consistent as the latter is a world of flux and flexibility to nature’s movements that would not be easily configured around contemporary neoliberal economics, nor would the other narrative that falls close to this extreme (*Reciprocal Stewardship* (NC)). As worlds defined by investment in the deployment of technology for efficient production systems, it was consistent for *Sharing through Sparing* (NN–NS) and *Arcology* (NN) to be positioned on this gradient with economic paradigms closer to green growth (Fig. 3c).

## Discussion

In this section, we discuss how the process of developing scenario skeletons and their corresponding illustrative narratives led to an improved understanding of what the Nature Futures Framework offers to the development of new scenarios for nature, both in terms of relativity and mechanisms for navigating between plural values, as well as in exploring the implications of diverse discussions on transformative change.

### Lessons from applying the Nature Futures Framework

We learned two key lessons from applying the Nature Futures Framework to the development of pluralistic scenarios related to relativity and ongoing debates about transformation.

#### Lesson 1: relativity

Developing illustrative narratives showed that the three value perspectives are connected to one another. As a result, the narratives needed to highlight not only the main differences between the corners, but also how they relate to each other (ESM3).

The original Nature Futures Framework (Fig. 1) depicts three coloured circles representing each value perspective (*Nature for Nature*, *Nature as Culture*, and *Nature for Societ*y). Some parts of each circle fall outside of the triangle, indicating future states based on individual nature value perspectives that are not desirable. Thus, a corner needs to offer the most extreme articulation of that value perspective that is still considered compatible with the other two value perspective corners, or of what is objectively ‘desirable’. For example, unhindered commercial exploitation of fisheries to meet societal needs could be considered an example of a ‘Nature for Society’ benefit, but infringes on the ‘Nature as Culture’ and ‘Nature for Nature’ value perspectives and so cannot fall within the triangle. Similarly, a ‘Nature for Nature’ intervention that seeks to eliminate people or their right to reproduction would also not lie within the triangle as it infringes on the ‘Nature for Society’ and ‘Nature as Culture’ perspectives.

Moving closer to one value perspective automatically means moving away from one of the others. It is important to capture this relativity consistently, not just in narrative description, but also in quantification. This relativity between extreme value perspectives ensures that emerging narratives are therefore inclusive, pluralistic, and representative of the many sustainable combinations that can take place within the triangle. The narratives described above depict one possible configuration. There are many other alternatives.

Exploring the edges of the Nature Futures Framework is a way to describe a set of extreme examples of ‘living in harmony with nature’, but that is also consistent with the three value perspectives. In essence, the edges of the triangle are the bounds of desirable futures (i.e. the space outside is not consistent with a ‘desirable’ space for nature) and everything within the framework can be a messy combination of all three value perspectives. This is important to emphasise as it does not limit the development of narratives around the edges of the triangle, but encourages a plural interpretation of what can happen inside, relative to each corner.

Even though the assessed gradients of protected areas and land sparing/sharing were to an extent similar, we think they served different functions in the discussion on how transformative change can be operationalised. The former is focused more on how to protect nature from people, whilst the other is largely a debate on how we produce food and fibre, either via sustainable intensification on land, leaving the rest to nature (land sparing), or an approach that includes biodiversity and nature within the food production process (land sharing). We also realised that this positioning was based on our linking these gradients to the original narratives and that other attempts at illustrative narratives could land in slightly different locations, but this is an important first attempt at mapping options and how they relate to each other. Considering the narrative positions on both of these gradients is also important for maintaining internal coherence of the narratives. For example, it would be inconsistent to have a world where protected areas are not part of the value discourse, but extreme land sparing is. The important outcome of this process is that it is possible to see how the richness of the narratives can open an important discussion of different combinations of transformative ideas, and how some are more aligned than others, but that they each have an element of differentiation, allowing for the full range of the triangle to be discussed.

#### Lesson 2: a space to hold ongoing debates on transformation

A core objective of our work was to situate desirable narratives for people and nature in ongoing discussions of futures for nature. Both the IPBES Global Assessment (Diaz et al. 2019; IPBES 2019) and the 5th Global Biodiversity Outlook (Secretariat of the Convention on Biological Diversity 2020) based on the IPBES Global Assessment, stress that internationally agreed sustainability goals cannot be achieved without transformative change, defined as “fundamental, system-wide reorganisation across technological, economic and social factors, including paradigms, goals and values”.

Yet, while the need for transformative change is starting to resonate in the global environmental governance arena, and some influential institutions are taking a stance on specific concepts, we are only just beginning to understand how systems and cultures can be actively transformed at the scale that is needed. What all these concepts have in common is that they are highly contested whilst under-explored. We argue that narratives of transformative change are needed as a starting point for consolidating these ideas into actual policies that may be applied. IPBES is currently working on an assessment of transformative change to come out in 2024 and within the scoping document is a need to find and assess visions and pathways of transformative change towards desirable futures (IPBES 2021). We offer these narratives based on the Nature Futures Framework as one example of what such transformed futures could look like.

Narratives of transformative change help people imagine and grasp the far-reaching implications of transformation and negotiate which transformed futures are considered just and desirable, and for whom (Wyborn et al. 2020). Evidence is starting to suggest that powerful frames and compelling narratives, more than rational arguments, influence positions on what is desirable and legitimate in the biodiversity and natural resource conservation agenda, therefore presenting alternative narratives can broaden policy approaches that are just and more inclusive of plural values (Louder and Wyborn 2020). New narratives created through the Nature Futures Framework will thus hopefully be able to challenge conventional modes of thinking, modelling and planning (Pereira et al. 2022). For many people, the prospect of profound change is not easy to accept and difficult to comprehend (Pereira et al. 2020b) and further, transformations are also not necessarily good for all; rather, they can have a ‘dark side’ that must also be acknowledged (Blythe et al. 2018). This is relevant within the discussion of potential positive’ tipping points for sustainability, many of which require complete reconfigurations of current systems, starting with what drives them in the first instance (e.g. consumption aspirations or economic growth targets). It may be possible to reach sustainable futures without such transformation in drivers and feedbacks, but as described above, we intentionally explored the more radical transformative changes that may be needed, such as the reconfiguration of economic and governance systems, as a starting point because we think it is important to be able to describe in a narrative format what futures could emerge from such interventions, so that more quantitative analysis can follow. Descriptions of transformation in the future may be instrumental to opening up discussions on transformative changes in the present, which is the intention of thinking about the future, or anticipatory governance, in the first place (Vervoort and Gupta 2018). With this in mind, it is important to note that the narratives we present here are not prescriptive; however, we offer these narratives as examples of what futures could look like if we were open to exploring diverse opportunity spaces. The possible narratives created by applying the Nature Futures Framework offer a starting point for people to realise how open the future really is if we are brave enough to make changes now. We also note that illustrative elements do not need to be specific to a certain Nature Futures Framework space; even in the same space of the triangle, different narratives can be written. The six narratives presented here can serve as guides for users to formulate additional and alternative narratives.

### Improving the development of illustrative narratives

The illustrative narratives presented in this paper are a step towards better inclusion of pluralistic values in nature futures. There may be multiple narratives that correspond to any given position within the Nature Futures Framework (hence the name ‘illustrative narratives’), reflecting how different cultures, societies, and geographies may translate the Nature Futures Framework into a local context and their future aspirations. It is important to note that the narratives we present here are all products of the participants’ viewpoints and interpretations of what could be considered ‘preferable’ from their own expertise and knowledge of key debates in the literature. These are therefore in no way intended to be blueprints towards a sustainable future. Rather, we acknowledge that each narrative is a product of negotiation between our own worldviews and understanding within a framework to ensure internal consistency and relationships between the six scenario skeletons and their associated specific value perspectives. These should therefore be read as illustrations of how a diverse group of people undertook the co-production of transformative visions of the future that they themselves consider to be plausible. In this regard, we consider that the proposed methodological approach would benefit from incorporating additional methodological steps to better track the underlying value assumptions contributed by each participant and therefore acknowledge and address biases.

We urge further development, including using other methods to develop alternative visions across scales and levels (Pereira et al. 2020a, b). One of the challenges in building narratives using the Nature Futures Framework is how to assess the desirability of a particular vision of the future. A general principle is that, at minimum, perspectives inside the framework should reflect an ambition to meet the Sustainable Development Goals, but the boundaries between desirable and undesirable futures are often context or place specific. Thus, deciding on the border between desirable and undesirable futures requires broad participation.

Models that can quantitatively evaluate nature and nature’s contributions to people could help critically evaluate whether futures described in qualitative narratives can be considered plausible and sustainable (for a review of such models used in recent global model intercomparison projects, see Weiskopf et al. 2022). However, the ability to quantify the narratives is impeded by the limitations of existing models, such as the ability to quantitatively evaluate the nexus between different global and local changes, telecoupling, and projections of transformative actions (IPBES 2022a, b, c). In this regard, improving existing models of nature and nature’s contributions to people and integrating them (Weiskopf et al. 2022) is important to facilitate the further development of the narratives in line with the Nature Futures Framework (Kim et al. in review).

### Further implementation and operationalisation of the NFF and illustrative narratives

Increased availability of new scenarios based on the Nature Futures Framework could help policy makers and other stakeholders to explore the pathways to achieve the 2050 Vision for Biodiversity of ‘Living in Harmony with Nature’ (CBD/COP/14/9) as well as the 2030 Agenda and its Sustainable Development Goals. To catalyse the development of such new scenarios, the task force drafted the preliminary methodological guidance on how to use the Nature Futures Framework for scenario development and analysis, building on a series of stakeholder consultations, including those attended by IPBES’ national focal points (IPBES 2022c). The latest work of the task force on the Nature Futures Framework and its methodological guidance can be found in IPBES document IPBES/9/INF/16 (IPBES 2022c).

The methodological guidance is still evolving and will be further updated. To further operationalise the Nature Futures Framework, knowledge gaps that need to be addressed by broader scientific communities include: (1) developing additional illustrative narratives as examples to showcase the plurality of scenario narratives that can be created using the Nature Futures Framework; (2) identifying and using indicators for the Nature Futures Framework that can be associated with different value perspectives; (3) addressing knowledge gaps in social-ecological feedbacks; and (4) advancing current modelling frameworks to facilitate the application of the Nature Futures Framework (IPBES 2022c).

In addition, the Nature Futures Framework could be used as a framework of scenario archetype analysis where future scenarios with similar underlying narratives, assumptions, and trends in drivers of change are grouped and located within the Nature Futures Framework considering their association with the three value perspectives (e.g. Kuiper et al. 2022; Quintero-Uribe et al. 2022). Also, the Nature Futures Framework can be used to assess underlying value perspectives and their implications within existing future scenarios, and modify them to become more nature positive. For example, there is an ongoing effort to modify the shared socioeconomic pathways (SSP) scenarios in the marine environment to assess the future of the fisheries sector (Cheung 2019; Cheung and Oyinlola 2019) by attributing one or multiple value perspectives of the Nature Futures Framework to the SSP scenarios and expanding the range of drivers, sectors, and policies (IPBES 2022c).

The mandate of the task force is not to do this work, but rather to catalyse such activities by the wider scientific community, including the formation of new research consortia and research projects that will create multi-scale (from local to global) Nature Futures Framework-based scenarios to be further developed and refined over the long term.

## Conclusions

In this paper, we illustrated how the Nature Futures Framework can be used to generate a diversity of scenario narratives that have nature at the heart of their storyline, and which are positive for nature and people. We provided a series of methodological steps to write these narratives, so the desirable futures captured by their storylines reflect their underlying value perspectives. Future work could strengthen the methodology by developing and integrating an additional process to better track each participant’s underlying values and associated assumptions, thus acknowledging and addressing biases. This would also facilitate the implementation of the proposed narratives across scales and levels by recognising areas, location or groups with associated underlying value assumptions.

This exercise also contributed to the ongoing effort to develop inclusive and pluralistic scenarios of desirable nature futures. We strongly believe that additional narratives could support the endeavour of expanding a new family of scenarios and models, so society can better map the actions, conditions and decisions needed to reach a future that represents everyone’s voice.

## Supplementary Information

Below is the link to the electronic supplementary material.

Supplementary file1 (XLSX 18 KB)

Supplementary file2 (DOCX 25 KB)

Supplementary file3 (DOCX 83 KB)

## Data Availability

Authors can confirm that all relevant data are included in the article and/or its supplementary information files.

## Footnotes

1 Though not repeated every time after “Nature Futures Framework”, it is understood that any mention of the framework implicitly includes this subtitle. Similarly, “Nature as Culture” implicitly refers to “Nature as Culture/One with Nature”.
