# Supplementary material for: Bringing the Nature Futures Framework to life: creating a set of illustrative narratives of nature futures
Source: Sustain Sci. 2023 May 4:1–20. Online ahead of print. doi: 10.1007/s11625-023-01316-1 (PMC10158677; doi:10.1007/s11625-023-01316-1)
Supplement: Supplementary file 2 — Supplementary file2 (DOCX 25 KB) [file 11625_2023_1316_MOESM2_ESM.docx]

**ANNEX 2 -** *Bringing the Nature Futures Framework to life: Creating a set of illustrative narratives of nature futures*

**Narrative name: Arcology (Nature for Nature)**

**Overview:** In this world, people respect and value all life on Earth intrinsically, and live in dense self-sustaining urban areas designed to minimise the role of humans in the biosphere and preserve nature in a wild state.

**Key words**: *high technology, pristine wilderness, knowledge economy, city-states, strictly protected area, ecological ethics, norms*

**[Economy, governance, cities, communities]**

All people live in high-tech cities that are very efficient in water use, recycling and which are designed to minimise pollution and the impact of resource extraction. In these city-states, the economy is mostly based on services and knowledge production, and the exchange of goods is possible with novel technologies. Systems of environmental-economic accounting have been extended to capture good quality of life, prosperity and sustainability. They are supported by monitoring systems which completely rely on innovative technologies. The scale and form of governance are dominated by global top-down UN style organisation of city-states and heavily supported through computer algorithms and Artificial Intelligence.

**[Infrastructure, energy, transport, water]**

Human infrastructures are exclusively limited to urban areas and optimised to be respectful to the environment and meet adequately the size of the population. Underground hyperloops and drones are used to connect cities to minimise anthropogenic impacts on nature. In the interests of efficiency, the production of energy is highly optimised at large scale to supply the cities-states and especially the data centres. All the energy is produced in a few high-tech clean and secure nuclear energy centres that have no significant impact on the natural world. In order to preserve water resources, the natural water cycle runs with little human intervention from local to global level.

**[Food, diet, agriculture, fisheries, aquaculture, land management, well-being]**

This world is characterised by an extreme land-sparing, with optimal use of space within cities and maximised production of services per area. Diets are perfectionized and smart healthcare is all around (e.g. remote health monitoring). For nutrition, technology has optimised meals and improved tastes and nutritional value, but not cultural diversity. The fresh food is limited to vertical urban gardens. All meat comes from laboratories or is ‘beyond burger’ tech. Wellbeing is primarily generated through virtual reality and supported by smart technological systems. The catches from marine and freshwater fisheries are limited as most ecosystems are set aside as protected areas. The aquaculture is dominated by seaweed and shellfish in scale that are well-within ecological capacity. There is a strong focus of ensuring co-benefits of aquaculture to enhance biodiversity. For ecological considerations, most of the aquaculture and agriculture production comes from lab-factories with low ecological footprint using high-technologic processes (e.g., microalgae production).

**[Megafauna, oceans, biodiversity use]**

In terms of biodiversity, the management of biotechnological processes is implemented for the delivery of ecosystem services within cities (e.g microbiotic systems for sanitising water quality, food production etc.). To ensure highly connected pristine areas and effectively preserve wilderness, all natural areas of interest for ecology, flora and fauna are strictly protected and all deep oceans are set as Marine Protected Areas with no take zone.

**[Trade, law-rights, education, policy]**

People voluntarily subscribe and conform to restrictions in their occupation of space. Ecology is the highest priority in constitutional law and by laws. Therefore, environmental education is integrated in all components of education. Policies enforce ecological laws and aim at facilitating their translation into the lives of people in megacities (or arcologies). Many of the regulations are meant to facilitate everyday life in these big, dense, compact, three dimensional social structures that try to fuse architecture with ecology. But the policies and regulations also establish political and diplomatic relations as well as norms of communication and behaviour outside the megacities, in the wild and in the ways the arcologies must exchange and live with each other. Urban security has precedence over personal privacy and this is reflected in preeminent policies and regulations laws. To rule this world, the laws are highly policed and normative.

**Narrative name: Sharing through sparing (Nature for Nature/ Nature for Society)**

**Overview:** People have a fairly strong use- orientation towards nature but also value and protect the self-regulating capacity of the biosphere as biodiversity and natural processes provide the resilience that enables humanity to stay within planetary boundaries. While sparing space for nature, remaining areas are used intensively but efficiently and sustainably.

**Keywords:** *resilience, protected areas, ecosystem services engineering, circular economy, sustainable optimization, urban rural flow. dynamic nature*

**[Economy, governance, cities, communities]**

The human world population is between 6-8 billion people. Most people live in urban areas, but there are people living and working in rural settlements in the countryside. Cities are nature-inclusive and redesigned to cope with a number of sustainability issues, including the regulation of water, air, and natural disasters, enabling sustainable optimization. New cities are developed on locations that are suitable from a sustainability perspective.

Governance is decentralised to a scale determined by urban areas and their directly surrounding ecosystem services provisioning landscapes, seascapes and protected areas. The vast areas that are allocated for large scale natural dynamics, often far away from cities, are governed through international treaties, to guarantee a stable functioning of the Biosphere.

The economy has shifted to a green, interconnected market economy that is decoupled from environmental impacts, including telecoupled ones. Environmental sustainability is thus prioritised over economic growth. The green economy is mostly biobased and centred on circularity, to minimise natural resources extraction and pollution. The private sector operates within a strict framework of rules, regulation, taxes and natural capital accounting.

The economy is driven by the generation of ecosystem services, through, for example, circular agriculture and green infrastructure. The economy is further powered by technological innovation, especially bio- and nano- and manufacturing technologies (such as 3D printing) promoting human wellbeing while marginalising environmental impacts, e.g. by reducing transport. Information and communication technologies generate large amounts of jobs that are decoupled from environmental impact. Digital innovations also make life easy and fun, reducing the need for travel. Tensions in communities that result from living in cities and living online are prevented through social innovation, with a large role for education.

**[Infrastructure, energy, transport, water]**

Where we don’t let nature run its course, we engineer with nature to optimise ecosystem services. Cities make great use of green infrastructure, ecosystem-based adaptation and nature-based solutions, with an important role for monitoring, algorithms and technology to manage and optimise co-benefits and (multi)functionality. Also in the more rural areas, green infrastructure is integrated with grey infrastructure (e.g. roads with bushes to protect against wind and erosion while promoting pollination) and meshed throughout the landscape along with agriculture and novel and high service-generating ecosystems.

All transport is clean, efficient, and fossil free with dominant use of public transport for short to medium trips and limited use of personal transportation.The global and long distance transportation is largely by air (clean blimp tech) and hyperloops.

Cities rely on local and regional energy generation from an optimal mix of renewables, primarily solar and geothermal energy, with ‘smart’ grids enabling shifting of energy supply in response to demands across the urban landscapes, which are connected to neighbouring cities. All water, heat and energy use systems are within a circular economy framework with local and regional production, management and use, operating efficiently and producing zero waste with wastewater, heat and other materials to be reused in a closed-loop.

**[Food, diet, agriculture, fisheries, aquaculture, land management, well-being]**

A combination of highly engineered nutrition balanced diets with a range of fresh local and seasonal produce leads to healthy life for urban dwellers. This diet draws on the “planetary health diet” that is optimal for people and the planet. Little meat is consumed from animal sources and alternative proteins are invented and made easily accessible at an affordable cost. This fairly efficient lifestyle is aided by the implementation of a circular economy, greatly reducing food waste and resulting in drastic change in human behaviour. Urban lifestyles around the world are fairly homogeneous, less materialistic and resource efficient as an important part of people's lives unfold online, reducing the demand for physical space (including those for agricultural and aquacultural production), sparing nature. The international travel for business and leisure is significantly reduced with increased use of remote meetings with continued advancement in technology. As a result, families spend more time together, also with nearby neighbours, while continuing the interaction with friends and colleagues in distance through the social media, overall enhancing the social cohesion locally as well as globally. People come to appreciate their everyday life where they are while appreciating distant wilderness and highly anticipating their recreational time in the space of urban-rural flow where humanity meets nature.

Agriculture generates high yields by making use of ecological principles to optimise ecosystem services. This is primarily achieved through nature inclusive circular agriculture, combined with smart technological systems to enable sustainable precision agriculture, with minimal inputs, making use of nature’s nutrient cycles while irrigation water and agricultural wastes are recycled and reused. Besides crop production and nature friendly livestock farming, agroforestry generates various ecosystem services, resulting in a multi-functional landscape. In practice this means that nature is not only far away in PA’s but also very much part of our production landscapes. Outdoor agriculture is complemented with high-tech greenhouse horticulture to further free up space for nature, minimise resource input and prevent pollution. In these systems all nutrient and water cycles are closed. In cities we find vertical horticulture, such as hydroponics and aeroponics. We see a locally integrated supply chain with efficient use of biomass and nutrients. Bio-refining is used to acquire raw materials such as sugars, proteins and lipids, providing the basis for biobased fuels and materials.

All marine areas are zoned for management, and where appropriate for optimised usage. In protected areas, fisheries, if any, are highly controlled and limited to a small number of species that provide the essential services for humans at an ecologically regeneratable level. Importantly, due to spill-over effects, Protect Areas (PAs) increase the production of stocks outside of the PA’s, enabling sustainable fisheries. Human activities without ecological impact occur in protected areas, however, with careful measure and monitoring over time. In all non-protected marine areas we find sustainable extraction of natural resources, which is heavily monitored and controlled to optimise production. There is very limited by-catch, all destructive fishing methods are prohibited and historically collapsed stocks are rebuilt.

Aquaculture, including seaweed, occurs in designated areas where nutrients and other chemicals are circulated as much as possible, and with strong environmental control and monitoring. It aims for maximum human benefits, with ‘high production, high protein’ species and focussing on nutrient-oriented production rather than on high economic value species, and new lower trophic level aquaculture is introduced (faster growing species, requiring less inputs) with high use of multi-trophic systems.

**[Megafauna, oceans, biodiversity use]**

Extensive areas of the planet are allocated for nature to be self-willed. Protected areas are the primary tool to enable wilderness and natural dynamics. We identify and protect those areas that matter most for safeguarding the self-regulating capacity of the biosphere. There is some management in those areas, to prevent anthropogenic modification and to enhance ecological resilience through spillover effects. Depending on their history, ecosystems have been restored through active stocking and reintroduction of extirpated species. Novel dynamics resulting from range-expanding species, driven by climate change, are allowed. Cascading and tele-connected disturbances from adjacent human activities are prevented. For example, dams in rivers are operated to maintain the natural flow regime. No predetermined percentage of PA’s is pursued; the percentage of protected area results from an evidence based asset.

People enjoy being in green spaces, both inside and outside cities, for recreational use and aesthetic pleasure. Yet, people generally do not experience a strong place-based, personal connection with nature, and as such, nature does not strongly contribute to identity or culture. Some activities are permitted in the protected areas of wilderness. Ecotourism is popular as people like to impress their social network with spectacular photographs, and because people want to see for themselves that nature is indeed protected for the purpose of providing global services. Journalism and nature documentaries report on the well-being and intrinsic values of nature, also serving educational purposes. Internationally agreed-upon scientific studies and expeditions seek use of the flourishing biodiversity in the protected areas. Biodiversity is a great source for technological advancement, using biomimicry and genetic resources.

**[Trade, law-rights, education, policy]**

Policies enforce the dual - conservationist and productivist/developmentalist - nature of the economic and legal structures in order to make them more balanced, and are connected throughout the land,the ocean and the cities. Land use and tenure regulations allow for productive use, with some protectionist limiting potential negative impacts on biodiversity and climate. International trade is moderate, enabling geographically optimised generation of provisioning services. The commodities are more local and regionally produced with completely non-fossil fuelled transportation systems for trade. Policies emphasise the primacy of global security over privacy rights with strict measures on protected areas and prioritising environmental and sustainable economy policies. There is a primacy of ecological and productivist legislation that is connected to the education curriculums and the social systems as a whole, maintained by information technology and Artificial Intelligence. People learn from early on to appreciate nature through education on instrumental values of nature through multiple benefits nature provides to people, intrinsic values through the role of biodiversity in sustaining healthy ecosystems and cultural value through the history and the evolution of human relationship with nature. The education is provided to people of all ages to ensure informed decision making and behavioural change.

An accounting system is in place to distribute costs and benefits related to the protection of nature among states, supported by a mediation and arbitration institute that resolves conflicts

**Narrative name: Optimising nature (Nature for Society)**

**Overview:** A highly connected world that shares knowledge and technology to maximise efficient and sustainable utilisation of nature’s contributions to people (including nutrition and food security), while ensuring maintenance of the key ecosystem functions that underlie them.

**Keywords:**Sustainable maximisation of nature’s contributions to people, social-ecological extensification; nutrition and food security

**[Economy, governance, cities, communities]**

The world is highly organised and regulated through top-down, centralised governance systems with a high degree of global cooperation. Governmental institutions work closely with the private sector to advocate evidence-based decision-making that ensures resources that are used efficiently and distributed equitably. This is implemented through policy targets that reflect the importance of maintaining ecosystems to support health, nutrition and food security, such as taxation that internalises environmental costs, regulations that promote informed lifestyle choices, laws that enforce the sustainable management of land/seascapes, and accessible reciprocal agreements for ecosystem services. Such policies are supported by significant investment in research, innovation and technology. This ensures governmental institutions, private sectors and wider society to be ecologically literate and consider the positive or negative impacts of decisions on biodiversity and nature’s contributions to people. Such technological innovations are co-developed between producers, researchers and industry to be respectful of nature, to make use of local biodiversity, and to assess and monitor optimal ways of utilising nature’s contributions to people. Principles of law and policies are also designed to respect and enhance cultural and biological diversity.

**[Infrastructure, energy, transport, water]**

Most people live in high tech cities that are designed to maximise the efficiency of resource use and to support the sustainable delivery of multiple contributions to people from nature, for example through pollination corridors, vegetated buildings and artificial wetlands. All cities in the world look similar and are highly connected through the transfer, sharing and trading of goods, services, knowledge and technology. Cities are also highly connected with surrounding clusters of rural settlements. Through the tight dependence and linkages to ecosystem services, urban and rural dwellers reconnect with nature, reconcile their interests and assist each other in improving quality of life in the cities and valuing the countryside. Society appreciates this “tamed” nature which stimulates innovative entrepreneurship across urban-rural landscapes and localised ecosystem services flows (e.g., closing the nitrogen cycle at the landscape scale). The growth of the cities and other settlements takes account of natural capital, focusing on nature-based options for securing long-term prosperity.

People use environmentally-friendly carbon neutral public transport for connectivity across the planet. This facilitates intermodal mobility between remote regions and urban-rural areas. This also reduces inequalities within and between urban and rural areas, and enhances liveability of rural areas.

Energy supply is from green/renewable sources of energy, decentralised but connected by highly efficient and ‘smart’ energy distribution grids. Energy utilities are highly efficient and energy security is high.

**[Food, diet, agriculture, fisheries, aquaculture, land management, well-being]**

Land used for production and resource extraction is planned and managed sustainably within a landscape matrix that supports biodiversity and ecosystem functioning, whilst delivering multiple nature’s contributions to people. People in the world rely on food produced from nature, with food production systems being highly efficient in both food production and conserving biodiversity, assisted by advanced technology. Large areas of land are used for crop and livestock production due to ecological extensification of agriculture, but management practices are biodiversity-friendly, avoiding excess nutrients from fertilisation and minimising waste. Genetically modified crops are socially accepted, ethical and widely used, permitting the production of food with low resources. Agricultural landscapes are diverse to deliver multiple ecosystem services, rather than simply food. This includes agro-forestry systems, mixed forestry systems, wetlands and connected mosaics of habitats to provide recreation and aesthetic value as well as supporting the resilience of biodiversity and ecosystems.

Almost all aquatic systems are used for food production from fisheries and aquaculture. Fishing operates in the high seas and Exclusive Economic Zones (EEZs). Fishing technology allows for precise extraction of biomass to maximise for ecosystem-level production with low biodiversity impacts, i.e. minimization of by-catch and ‘balanced harvest’ strategies. New technology and incentive systems allow for efficient and effective control, monitoring and regulation. Similar to agriculture, ecological extensification happens in aquaculture in both freshwater and marine systems, e.g., offshore aquaculture technologies allow for farming of aquatic organisms in the high seas. New multi-trophic aquaculture techniques and systems are developed that allow for efficient utilisation of nutrients with minimum ecological impacts. There is no need for fish meals and fish oils inputs; those fish instead are used directly for human consumption.

Food systems are global and fully integrated. Large international corporations together with effective government regulations ensure that production systems are highly efficient with low ecological impacts. Food processing technology is advanced, resulting in almost 100% use of biomass and nutrients from food products. Globalisation and high equity results in everyone having access to a healthy and sufficient diet with a similar level of food diversity. The ecologically literate population has a high awareness of the consequences of lifestyle choices and thus has relatively low consumption and a generally low ecological footprint. This eases potential trade-offs between land use for food production and habitat modification.

People in the world enjoy water security through highly managed use of water resources. New knowledge and technology allows for precise and effective allocation of water resources to maximise its benefits to people, whilst ensuring environmental flows to support aquatic ecosystems. This efficient management of water resources, including ensuring a high level of water quality, is through public-private partnership and allocation of use rights regulated by top-down water governance systems.

Other non-food extractive uses of natural resources, such as energy production and mining, take place on land and at sea. The use of knowledge, data, new technology and strong international and national governance and cooperations ensure accurate environmental impact assessments. Negative consequences on the environment, particularly those that would affect key ecological functions to support human benefits are avoided.

**[Megafauna, oceans, biodiversity use]**

As most land and sea is focused on delivering nature’s contributions to people, there are limited protected areas. Those that do exist are designated to protect key ecological functions that are essential for supporting ecosystem services. Nevertheless, land/seascapes are managed in a biodiversity-friendly manner so biodiversity loss is low. However, limited losses of biodiversity and modification of landscapes are considered socially acceptable so long as they do not adversely affect the long-term delivery of multiple ecosystem services.

**[Trade, law-rights, education, policy]**

People are highly educated in both urban and rural areas - with strong investment in engineering to support the world with high efficiency of utilisation of ecosystem services and maintenance of ecosystem functions. High awareness of the importance of ecosystems’ contributions to health, nutrition and food security helps conservation of nature’s functioning (link to education and consumer awareness programs). Full recognition of women’s role contribution and knowledge in the sustainable use and conservation of biodiversity.

**Narrative name: Innovative commons (Nature for Society/Nature as Culture)**

**Overview:** people have built a world of innovative ecological commons and live in interconnected green-blue cities and rural settlements across landscapes in all - or most - world regions

**Keywords:** Collaborative commons, community-based management, sustainable intensification, mosaic landscapes, land sharing, technology, blue-green infrastructure.

**[Economy, governance, cities, communities]**

This is a world where nature is lived as a culture that responds to the needs of people and communities. This is made possible by a thriving blue-green social economy (i.e a green economy mostly reliant on circular principles and local solutions) that is interconnected through moderately intense, equitable trade of goods and through the collaborative commons and peer to peer networks. The social economy is generative and regenerative: it does not just use markets but creates new economic value through diverse value streams (e.g. domestic labour and community work, or contributory creations over the Internet) and circular and ecological processes breaking away from past profit-maximising exploitative relations. A wide diversity of socially oriented organisations (e.g cooperatives, associations, social enterprises, community-based and integrated landscape initiatives) and institutions give shape to, and benefit from the social nature of the economy.

In such a world, governance is polycentric and globally decentralised and it links urban nodes to well connected and coordinated rural cultural groups in and across countries and regions. Global governance is based on the power of regions and a global balance of equity between strong, autonomous world regions. It strengthens a global movement for regional, decentralised, socially-oriented economic systems that ensure that benefits do not accrue only to powerful actors.

Blue-Green cities (medium to large) are designed around community-friendly ecological principles to deliver social services - from spiritual gardens to universal clean water. They link up with rural settlements to form a network of interconnected semi-autonomous entities across the landscape. Advanced nature-based technologies are shared and High tech is balanced by the social, decentralised nature of the digital commons and by P2P (peer to peer) networks that link virtual communities with “real” rural and urban communities of practice. A strong identity to culture and place is diffused and shared throughout community-based networks and collaborative commons.

**[Infrastructure, energy, transport, water]**

Transport is multimodal, using public as well as individual means of transport by land, air, sea and river, but it is based on innovative, low impact technologies that connect people and goods locally and regionally. Transportation effectively supports an environmentally sustainable trading system.

The energy system is a collaborative energy regime in which millions of people produce their own renewable energy (solar, wind, thermal, etc.) with the help of medium-size and micro–power plants, as well as, advanced storage technology, including hydrogen, to store intermittent energy. Energy is loaded and stored by buildings (homes, offices, universities, hospitals, factories, etc) and is deployed throughout existing infrastructure. Excess energy is freely traded over the Energy Internet (EI) by autonomous energy producing and consuming communities. The system is a highly decentralised, connected energy network, with energy trading happening over the Internet of Things (IoT) mainly through sharing and collaborative principles. Commoning, reciprocal credit, and barter trade are essential to trade over the EI and prevail over old supply and demand market pricing.

Water systems are managed and modified where necessary to satisfy the needs of humans, taking into account their cultural traditions along with considerations of human and natural well-being.

The overall ecological infrastructure is based on a combination of traditional practices and novel technologies, including symbiotic bio-organisms adapted to productive uses, and it targets the conservation of culturally significant species in community conserved areas and co-managed landscapes, along with charismatic megafauna for tourism in protected areas representing no more than 14% of land areas, mainly in the form of community conserved areas. In the oceans, people practice ecological restoration and management of forms of oceanic resources backed up by strengthened local processing and transformation in order to sustain local economies and maritime cultures and trade (Blue economy: equity and protection of coastal communities) as well as community-based coastal tourism.

**[Food, diet, agriculture, fisheries, aquaculture, land management, well-being]**

Collaborative land management is practised where local and indigenous communities and public and private institutions implement co-designed strategies for sustainable land use. The strategies favour ecological intensification that requires optimal management of biodiversity and ecosystem functions using knowledge-intensive (i.e. combining ethnoecological and scientific knowledge) and participatory (i.e. involving communities and institutions) processes. Land is managed through a mix of land-sharing and land-saving. Traditional practices for productivity and culture are implemented in rural areas, and between middle-size cities. Ecoagriculture is dominant in mosaic landscapes, which are managed in a way that integrates agriculture with forests and other land uses on a sustainable basis. Ecological intensification of agriculture enables land-saving social innovations based on blended ethnoecological and scientific knowledge. Monocultures are rare to non-existent. Production is intimately related to the enlarged reproduction of diverse food-related identities and cultures that are shared within and across landscapes, countries and regions.

Open fisheries are dominated by small-scale fisheries and coastal communities producing and trading mostly for domestic local and urban uses, for both traditional and economic benefits. Community-operated cooperatives run the supply chains (both locally and internationally). Fisheries are effectively monitored and controlled by these cooperatives in link with national science and policy institutions. The priority is to support the enlarged reproduction of diverse local cultures and communities within and across the nation.

Aquaculture has become a major source of fish supply, representing nearly a quarter of the world’s fresh fish supply. This happens in parallel with a reduction of fishing effort in open fisheries and the generalisation of more sustainable fish harvests. Adoption of new aquaculture techniques, strongly integrated with agriculture, allows for conservative land usage, along with increased production of a mix of traditional species. This generates economic benefits in support of local communities and livelihood.

Diets and local consumption are mainly based on diverse, pluricultural traditional foods, closely tied to culture and landscape. There is an innovative use of technology to allow for different foods to be traded and appreciated elsewhere-anywhere.

**[Megafauna, oceans, biodiversity use]**

Traditional and local ecological knowledge for the use of biodiversity is implemented in rural areas and within green cities (e.g. medicinal use). Communities use their local and traditional knowledge, and technology, to expand the use of biodiversity and also to enhance understanding and recognition for various cultures among communities. Natural biological resources (e.g. natural products and nutraceuticals) are widely accessed and sustainably used, and benefits arising out of utilisation of genetic resources are shared by all social categories.

**[Trade, law-rights, education, policy]**

An important part of policy is to secure people's free and secured access to a 10G-1Q worldwide web through advanced types of open source or creative commons licences. Policies incentivize knowledge-policy integrations that facilitate land-saving, resource-saving innovations, allowing for more ecological productive processes. Formal regulations are limited by the primacy of citizen networks and informal agreements, and by heavy investments in the education system, with an early emphasis on community and nature, culture and history, human and natural diversity and complementarity

Networks of citizen forums form a strong basis for social ecological awareness and are actively engaged with political processes and law enforcement. Laws emphasise community rights, voluntary engagement but also economic cooperation through the commons and the productivity of ecoagriculture and other production systems.

**Narrative name: Reciprocal stewardship (Nature as Culture)**

**Overview:** This vision illustrates a world where values of reciprocity and harmony drive humans’ relationships with nature at all levels of human organisation. Biological and cultural diversity are co-conserved and co-managed across a wide range of interconnected bio-cultural systems.

**Keywords:** Community-based,holistic,system approaches, sense of place, stewardship, identity. Heritage. Relational values.

**[Economy, governance, cities, communities]**

This vision is supported through governance processes that take precedence at the scale of self-determined jurisdictions, resulting in a rich diversity of governance systems. The latter recognizes indigenous people’s sovereignty over their lands and knowledge systems, and captures the local identity and needs of local communities. The wide variety of governance systems is challenging to manage in an integrated way, since these are very context-dependent and self-determined (bottom up). Nevertheless, the shared fundamental values towards nature facilitates cross-system interactions. Horizontal governance systems work at the level of small cities, interconnected with a patchwork of autonomous rural settlements. Communities live connected to nature through evolved and high-tech traditional practices, thus creating a resilient and functional continuous bio-cultural landscape. Here, ‘high-tech’ refers to processes and materials that were cleverly thought and designed to fuel balanced human-nature relationships. High-tech can involve noble materials and rustic processes, but always in favour of promoting cultural and social engagement with nature. Within communities the sense of family is strong and these are interconnected. Elders are recognized and respected.

**[Infrastructure, energy, transport, water]**

Infrastructure is designed to handle small-scale processes, activities and community needs; capturing the local context. For instance, transport is based on multi-modal travel (i.e. bikes, horses, chariots, cars), used for very specific situations since it is costly and not totally environmentally friendly. This infrastructure enables trade of local food produce and cultural brands. Infrastructure for energy is also at a small scale; each house/building produces its own energy from different sources of energy (e.g. all buildings have their own wind turbine and/or solar panels). Trade of energy is possible among settlements. The design of freshwater system infrastructures stems from the rooted socio-cultural value towards this “living system”. This means that the rest of infrastructures have adapted to fit freshwater systems in a holistic manner.

**[Food, diet, agriculture, fisheries, aquaculture, land management, well-being]**

Food production is at small scale and for local consumption, based on the cropping and harvesting of a wide diversity of edible species. Food consumption patterns are highly seasonal, and the cultural significance of eating is a core value.

The maintenance of indigenous and traditional practices is fundamental within productive systems. Small-scale and community-based management predominates in fishing, farming and agriculture. Traditional aquaculture (e.g. clam gardening, mixed agriculture-fish pond) complements local food production, as well as the harvesting of wild relatives. These types of productivity systems are only attained with strong collaboration within families and communities, which is fostered by a strong sense of place, spiritual connection with nature - all contributing to health and wellbeing.

This way of functioning results in a highly heterogeneous and functional landscape. Land-sharing is actively practised. Yield gaps are not a social problem because they are widely acknowledged as an inherent result of this kind of productive system. The strong sense of place, cultural identity, and mental and spiritual connection with nature, all contribute to health and wellbeing.

**[Megafauna, oceans, biodiversity use]**

The persistence of terrestrial and marine biodiversity, as well as of ecological processes, are secured through traditional stewardship-oriented scape management. Culturally significant species are conserved at the expense of others in co-managed scapes (no-PAs). Deep relational values with nature have established a fundamental understanding about the complexity of ecosystems and permit practical and integrated conservation of land/seascapes and species. Respectful dialogue between indigenous and local knowledge and science facilitates the use of traditional/indigenous knowledge to manage community resource use, and maintain cultural identity through sustainable consumption of wild species, or if welcomed, openness for cultural change in relation to consumption of threatened species.

Food, meat, fibres and materials are produced and harvested through traditional and sustainable methods, which are deeply embedded in local identities and cultures. Wild relatives are protected and its cultural value is widely acknowledged in societies.

**[Trade, law-rights, education, policy]**

Local policing is done with a lot of citizen involvement. Laws emphasise community rights, socialisation is high, and enforcement is done mainly through social networks, voluntary engagements, and in conformity with social norms. Policies and regulations aim at reinforcing the cultural fusion of ecological dynamics with community histories and priorities. Much of this effort is directed toward the educational system and social institutions, as well as the sustainability of the food system, including product extraction from the wild to satisfy human needs related to health, nutrition, cosmetics, or nutraceuticals.

The development of new metrics such as a new Gross National Happiness (e.g Bhutan), are vital to guide regional and international collaborations. Such collaborations are led by an international entity that has attained no net economic growth, and promoted a wellbeing economy based on local cultural specificities, (e.g eco-tourism).

There is no net economic growth. There is an economic exchange that focuses on the social value of things rather than their monetary value. Traditional economy in the sense of low-impact tech, tailored to very specific local needs. Technology is advanced but it is specialised for the functional that reinforce interpersonal relations and cultural connectivity with and through nature.

**Narrative name: Dynamic Natures (Nature as Culture/Nature for Nature)**

**Overview:** Dynamic, connected and biodiverse ecosystems are valued to allow traditional socio-cultural reproduction, spiritual values and connections to be re-established and new ones to be shaped. Society accommodates the dynamism of nature through both traditional and innovative lifestyles that takes into consideration cultural heritage and traditional ecological knowledge.

**Keywords:** *dynamic nature, traditional knowledge, innovative lifestyles, natural-cultural landscapes, self-sufficiency.*

**[Economy, governance, cities, communities]**

Human societies respect, value, and accommodate the dynamism of nature through both traditional and innovative lifestyles that take into consideration cultural heritage and traditional ecological knowledge. Healthy and biodiverse ecosystems enable traditional socio-cultural reproduction, spiritual values and connections to be re-established and new ones to be shaped.

This is a polycentric world of nested social-ecological systems governed by autarchic communities that are defined by their ecosystem rather than social boundaries (e.g watersheds, biomes etc). There are therefore no more nations states. Whilst there is limited global trade and resource sharing, there is a high level of cooperation between autarchic units in order to ensure global compliance with global environmental legislation for the freedom of movement of ALL species and for regional knowledge sharing and governance. Adaptive and dynamic land and water management practices account for the season and geography, and are very context-dependent and flexible.

**[Infrastructure, energy, transport, water]**

Water is identified as key to life resulting in a strong demand for the recognition and restoration of the socio-cultural role of rivers as living systems. These now have a legal standing, together with the environment as a whole. Rivers flow freely without impediment. People use local and less globalised resources within circular economies, and focus on the creation of dynamic ecological infrastructure with much fewer roads. This means there are no more dams or large-scale permanent inorganic infrastructure.

Human settlements are dynamic and adapted to the movements of nature while being flexible (some nomadic) to ecological shifts using innovative technologies such as floating houses. Adaptive, dynamic transport uses tides, wind power and new technology that is able to capture these natural forces, building on traditional knowledge (e.g. Polynesian/pacific island boats). To consume the minimum amount of resources, buildings are well integrated to their environment (in some instances being embedded within hills or cave dwellings). There is a community-driven demand for decentralised, local control over resources. Energy is produced from renewable materials. More effort is put into reducing energy consumption than producing it and so each community is energy secure and self-sufficient.

**[Food, diet, agriculture, fisheries, aquaculture, land management, well-being]**

Diets are diverse and seasonal, based on what can be grown locally and ecologically without monocultures. Food production relies on harvesting from traditional production systems that have evolved with and are adapted to ecological dynamics. Pastoralism and gathering of wild fruits and cultivation of short season crops are preferred over permanent agricultural structures. Agro-ecological landscapes, including agro-forestry are linked with traditional technologies (e.g. terracing). Fishing is limited to traditional grounds within EEZs, dominated by small-scale fisheries, with limited production of high-value food products that are primarily to support local communities and livelihoods. Community-based and ecosystem-based fisheries management are in place. Traditional multi-trophic eco-aquaculture systems as well as aquaculture are tightly integrated with agricultural systems.

**[Megafauna, oceans, biodiversity use]**

There are no protected areas per se, but rather entire ecological communities are protected in situ (land and sea) as this is especially important for the conservation of migratory species. Many species are indirectly conserved as humans make way for nature and benefit from connected dynamic ecosystems, including novel dynamic conservation areas within a broader cultural landscape that recognises the rights of local communities. Ecological laws are enforced by community monitoring through citizen networks using the latest in drone and other non-invasive technologies. Strong environmental education is developed based on different cultural/traditional backgrounds and context. Every human feels connected to their community and values of reciprocity, harmony and relationality which characterise humans’ relationship with nature.
