# Supplementary material for: Bringing the Nature Futures Framework to life: creating a set of illustrative narratives of nature futures
Source: Sustain Sci. 2023 May 4:1–20. Online ahead of print. doi: 10.1007/s11625-023-01316-1 (PMC10158677; doi:10.1007/s11625-023-01316-1)
Supplement: Supplementary file 3 — Supplementary file3 (DOCX 83 KB) [file 11625_2023_1316_MOESM3_ESM.docx]

**ANNEX 3 -** *Bringing the Nature Futures Framework to life: Creating a set of illustrative narratives of nature futures*

**This document presents the resulting tables from the paired comparison exercise between narratives. To facilitate the comparison exercise, participants assigned a number to each of the six characterised scenario skeletons (Figure A1). The numbering starts from the top of the triangle, and clockwise, it begins with the three corners and extreme nature perspective. Then it continues with the middle points. Note that the scenario skeleton corresponding to number five, at the centre of the triangle was eliminated (details in Methods).**

**
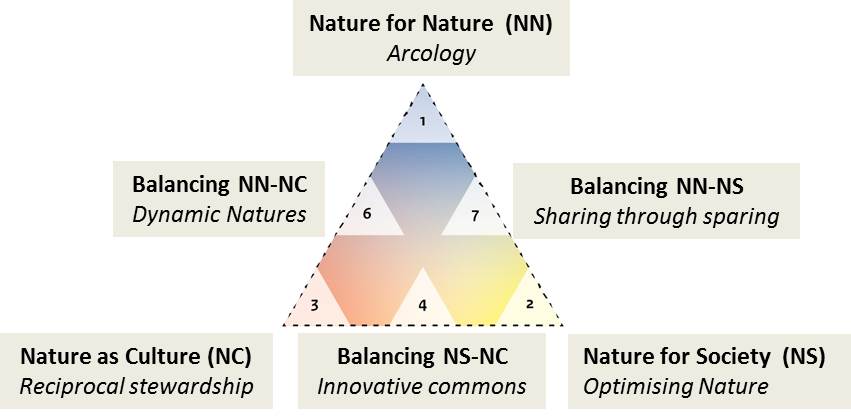
**

**Figure A1.** Distribution of six scenario skeletons across the triangular space, with their corresponding nature perspectives designated number to perform comparison exercises during the workshop.

**PAIRED COMPARISON BETWEEN ‘ARCOLOGY’ (1) NARRATIVE AND OTHERS**

1. **Arcology (NN or 1) vs Optimising nature (NS or 2)**

| **SIMILARITIES** | **DIFFERENCES** |
| --- | --- |
| **Governance, cities and communities** | |
| - People live in high-tech cities - Monitoring systems which completely rely on innovative technologies. - Global top-down governance. | - In 2, many people live in rural areas but not in 1. |
| **Infrastructure, energy and transport** | |
| - Populations are connected by high technology and communication/transportation systems. - Utilisation of clean energy. | - Human infrastructures are exclusively limited to urban areas in 1, but more dispersed and extensive, including rural areas in 2. - Focuses strongly on renewable energy from ecosystems in 2, while 1 also has focus on nuclear energy centres. |
| **Food, Diet, Agriculture, Fisheries, Aquaculture, land management, well being** | |
| - Extreme land-sparing - Availability of healthy and sufficient food with technology innovation. | - Land-sharing - More diversity of food in 2 than in 1. |
| **Megafauna, oceans and conservation** | |
|  | - Strong preservation of biodiversity in 1 but very limited protected area in 2. 2 also allows for biodiversity loss. |
| **Trade, law-rights, education, policy** | |
| - High education | - Education focuses on conservation in 1, but engineering on sustainable use of ecosystem services in 2. |

1. **Arcology (NN or 1) vs Reciprocal stewardship (NC or 3)**

| **SIMILARITIES** | **DIFFERENCES** |
| --- | --- |
| **Governance, cities and communities** | |
|  | - In 1 governance is strongly top-down (very centralised), while in *3* it is strongly bottom-up and horizontal. - In 1 there are no rural areas, at all, while in *3* landscapes are made up of rural patchy areas. - In 1 everyone lives in cities, while *3* people live in rural areas. - In 1 families are really small, they maybe even disappeared and therefore the relationship within “urban communities” is very horizontal. In *3*, families are big and very interconnected within rural settlements. |
| **Infrastructure, energy and transport** | |
| - Both 1 and 3 use innovative energy systems. | - In 1 there is no infrastructure outside of cities, while in *Rs* there this is integrated into the landscape. - In Arcology energy is not renewable (nuclear) and highly centralised, while in 3 completely renewable and decentralised. - In 1 transport systems are focused on speed, efficiency, trade and mobility. In 3 transport systems focus on social connectivity. - In 3 transport systems are highly multimodal, while in 1 they are not. - In 1 water systems are closed and use innovative systems (i.e. microbiological processes) for sanitising. In 1 hydrological systems are “wild” and not intervened. In 3 hydrological systems are intervened throughout the landscape, but at watershed level with small level extractions. |
| **Food, Diet, Agriculture, Fisheries, Aquaculture, land management, well being** | |
|  | - In 1 agricultural diversity is much lower than in 3, and fresh food is very limited (i.e. produced in labs and house roofs). - In 1 food production isn't seasonal,while in 3 is highly seasonal. - In 1 diet is driven by technical and resource capacity, while in 3 is driven by cultural heritage and value. - In 1 fishing and aquaculture are restricted to highly controlled MPAs, while in 3 fisheries are small-scale and community-based managed. - In 1 land-sparing is maximised, while in 3 land-sharing is maximised. |
| **Megafauna, oceans and conservation** | |
|  | - In 1 there are mega PAs, untouched natures, while in 3 there aren’t PAs and biodiversity protection is achieved by the co-management of the landscape. A similar protection system is applied to oceans. - In 1 biodiversity use is purely instrumental; its use stems from the benefits biodiversity provides. However, in 3, the use of biodiversity stems from a relational value and reciprocal relationship. |
| **Trade, law-rights, education, policy** | |
| - In both visions there is trade of knowledge and services. - In both visions there is an anarchical system of power, in which people (in 1) and communities (in 3) are self organised. | - In 3 the trade of goods is more frequent than in 1, but it focuses on the social value of things rather than their monetary value. - In 3 laws emphasise community rights and socialisation is high. However, in 1 law focus on the optimisation and operationalization of the metropolis. - In 3 laws stem from relational and cultural fulfilment, while in 1 stem from ecological efficiency. - In 3 education emphasises the interdependence between communities-people and nature, while in 1 social organisation within cities. |

1. **Arcology (NN or 1) vs Innovative commons (NS-NC or 4)**

| **SIMILARITIES** | **DIFFERENCES** |
| --- | --- |
| **Governance, cities and communities** | |
|  | - 1 has top-down UN-Style centralised governance, while in 4, governance is a decentralised and polycentric - 1 is based on megacities and arcologies while 4 is based on a networks of well-connected medium-size to small cities and extensive rural settlements |
| **Infrastructure, energy and transport** | |
|  | - 4 is based on renewable energy, well-connected ‘smart’ grid, while 1 is powered clean nuclear energy t - There is an extensive transportation system In 4, while in 1 there is no extensive transportation infrastructure out of the cities |
| **Food, Diet, Agriculture, Fisheries, Aquaculture, land management, well being** | |
| - Both 1and 4 foster innovation | - In 1 lab-produced synthetic food predominate, against food produced and collected in largely rural landscapes in 4 - In 1, fishing is very limited and controlled, while in 4 it is fairly extensive |
| **Megafauna, oceans and conservation** | |
|  | - Most of the planet is protected in 1 (70%), while in 4 it is a low proportion (<14%). - In 1, the fundamental aim is to preserve wild nature in pristine state, while in 4 protected areas are primarily designed to safeguard biological and cultural heritage |
| **Trade, law-rights, education, policy** | |
|  | - In 1, laws are highly policed, normative and top-down to enforce strong restrictions on families and access to the wild. - In 4 the laws and policies are highly deliberative and enforcement is heavily dependent upon citizen forums and community engagement. |

1. **Arcology (NN or 1) vs Dynamics natures (NN-NC or 6)**

| **SIMILARITIES** | **DIFFERENCES** |
| --- | --- |
| **Governance, cities and communities** | |
| - High education and strong environmental education. | - Top-down governance vs Decentralised - In 6, governance is polycentric with autonomy communities, while in 1 it is top-down centralised governance - In 1 people live in high-tech cities while in 6, cities are dynamic, flexible and adapted to nature ‘nomadic cities. - In 6, community-driven demand for local control over resources, connecting to the value of recipicality, harmony and relationality while in 1, communities are strictly urban. - In 1, globalised, highly integrated economy while in 6, it focuses on the local economy. - In 1, high-tech economy is based on services, digital economy, exchange of goods between cities possible with novel technologies, economy of knowledge in 6 it has strong environmental education that is based on traditional and cultural background. |
| **Infrastructure, energy and transport** | |
|  | - In 1 nuclear centralised clean energy and in 6 renewable, autonomous systems. - There is no infra-structure outside cities in 1, while in 6 we find dynamic housing embedded (some nomadic). - Underground hyperloops and drones are used to connect cities to minimise anthropogenic impacts on nature in 1 and in 6, dynamic transport that uses tides, wind power and new technology that really is able to capture these natural forces, building on traditional knowledge (e.g. Polynesian/pacific island boats). Travel by air and sea is enhanced. |
| **Food, Diet, Agriculture, Fisheries, Aquaculture, land management, well being** | |
| - Use of aquatic ecosystems for food resources. | - In 1, lab food development (no fresh food) while in 6, harvest relies on traditional systems that have evolved and adapted to ecological dynamics (agroforestry, permaculture, traditional technology). - In 6 fishing small-scale for local communities and livelihoods while in Arcology very limited catches because of MPAs.. - In 6, aquaculture focuses on mixed aquatic-land farms, however in 1 the production is restricted to specific areas and dominated by seaweed and shellfish in scale that are well-within ecological capacity. |
| **Megafauna, oceans and conservation** | |
|  | - In 6 Dynamic protected landscape but in 2 all protected; - In 6 recognize community and traditional rights while in 1 conservation measures are top-down controlled (highly connected 'pristine' areas, maximised for nature) |
| **Trade, law-rights, education, policy** | |
| - Laws and regulations are based on ecology - High education and strong environmental education. | In 1, globalised, highly integrated economy while in 6, it focuses on the local economy.  In 1, high-tech economy is based on services, digital economy, exchange of goods between cities possible with novel technologies, economy of knowledge in 6 it has strong environmental education that is based on traditional and cultural background. |

1. **Arcology (NN or 1) vs Sharing through sparing (NN-NS or 7)**

| **SIMILARITIES** | **DIFFERENCES** |
| --- | --- |
| **Governance, cities and communities** | |
| - Importance of AI and algorithms - Highly urbanised - Cities located in appropriate areas | - Top-down governance in 1 vs decentralised in 7 |
| **Infrastructure, energy and transport** | |
| - Strong focus on non disruptive transport - Natural dynamics and cycles (e.g. water, elements) in areas allocated for nature | - In 7, more disruptive and especially distributed infrastructure and transport happens in areas allocated for human use - Absence of dams in 1 - Micro-grids at the landscape level vs city level |
| **Food, Diet, Agriculture, Fisheries, Aquaculture, land management, well being** | |
| - Marine ecosystems are heavily monitored and controlled - The aquaculture production is restricted to specific areas - Optimal use of urban space - Urban gardens are used to produce fresh food | - In 7 all aquatic areas are zoned to maximise production however in 1 most ecosystems are set as Marine protected areas. - Multi-trophic systems in 7 vs seaweed and shellfish aquaculture |
| **Megafauna, oceans and conservation** | |
| - Extensive protected areas. | - Some activities are permitted in protected areas in 7 however the access is very limited to these areas |
| **Trade, law-rights, education, policy** | |
| - Primacy of ecological legislation - The importance of environmental education | - Urban security has precedence over personal privacy |

**PAIRED COMPARISON BETWEEN ‘SHARING THROUGH SPARING’ NARRATIVE AND OTHERS**

1. **Sharing through sparing (NN-NS or 7) vs Arcology (NN or 1)**

| ***SIMILARITIES*** | ***DIFFERENCES*** |
| --- | --- |
| **Economy, governance, cities, communities** | |
| - Importance of AI and algorithms - Highly urbanised - Cities located in appropriate areas | - Top-down governance in 1 vs Decentralised in 7 |
| **Infrastructure, energy, transport, water** | |
| - Strong focus on non disruptive transport - Natural dynamics and cycles (e.g. water, elements) in areas allocated for nature | - In 7, more disruptive and especially distributed infrastructure and transport happens in areas allocated for human use - Absence of dams in 1 - Micro-grids at the landscape level vs city level |
| **Food, diet, agriculture, fisheries, aquaculture, land management, well-being** | |
| - Marine ecosystems are heavily monitored and controlled - The aquaculture production is restricted to specific areas - Optimal use of urban space - Urban gardens are used to produce fresh food | - In 7 all aquatic area are zoned to maximise production however in 1 most ecosystems are set as Marine protected areas. - Multi-trophic systems in 7 vs seaweed and shellfish aquaculture |
| **Megafauna, oceans, biodiversity use** | |
| - Extensive protected areas | - Some activities are permitted in protected areas in 7 however the access is very limited to these areas |
| **Trade, law-rights, education, policy** | |
| - Primacy of ecological legislation - The importance of environmental education | - Urban security has precedence over personal privacy |

1. **Sharing through sparing (NN-NS or 7) vs Optimising nature (NS or 2)**

| ***SIMILARITIES*** | ***DIFFERENCES*** |
| --- | --- |
| **Economy, governance, cities, communities** | |
| - Societies have a use-orientation towards nature (*but the placed importance on the role of ecosystems differently between 7 and 2*) - NCPs to health are valued in both scenarios | - Top-down, centralised governance systems in 2 vs decentralised in 7 - Society place importance on material contributions in 2 vs. societies value regulating contributions in 7 - Law and policies respect not only biodiversity and cultural diversity in 2 vs food production and nature conservation are the main focus in 7   Missing in narrative 7 (or something we can learn from 2)   - A high degree of global cooperation in 2 vs not clear in 7 - Governmental institutions work closely with the private sector in 2 vs the role of the private sector not clear in 7 |
| **Infrastructure, energy, transport, water** | |
| - Extensive use of green infrastructures and NBSs - Dwellers are connected with nature in and around cities (and rural areas in 2) - Use of carbon-neutral (in 2) or carbon-free (in 7) transportation - Energy sources are renewables (primary solar in 7 vs. not clear in 2) - Distributed energy supply with smart grids | - Nature is tamed in 2 vs. wilderness is conserved in 7 vs. - Nature is taken into account for securing long term prosperity in 2 vs. nature provides resilience that enables humanity to stay safely within the planetary boundaries in 7   Missing in narrative 7 (or something we can learn from 2)   - Clusters of rural settlements surround cities in 2 vs. rural settlements are not mentioned in 7 - Transportation network reduces inequalities within and between urban and rural areas in 2 vs. inequalities not mentioned in 7 |
| **Food, diet, agriculture, fisheries, aquaculture, land management, well-being** | |
| - Highly efficient production with the use of advanced technology and smart tech to balance production and conservation - Global food supply chain | - Large areas of land are used for crop and livestock production due to ecological extensification of agriculture in 2 vs. highly efficient food production in urban areas with optimised yield with minimal inputs, implying less land used for crop production, and livestock production is less with little animal meat is consumed in 7 - Almost all aquatic systems are used for food production from fisheries and aquaculture in 2 vs. fishing and aquaculture is controlled and take place in limited areas - Global food supply chain and large international corporations implied active international trade in 2 vs. there is moderate international trade through the supply chain is globally integrated in 7 - Other non-food extractive uses of natural resources, such as energy production and mining, take place on land and at sea in 2 vs. energy extraction (not clear about mining) is limited in 7 as the power generation rely on the mixed of renewables, primarily solar   Missing in narrative 7   - Genetically modified crops are socially accepted in 2 - Agricultural landscapes are diverse to deliver multiple NCPs. in 2 vs. not clearly mentioned in 7 - Fishing technologies and aquaculture techniques are mentioned in 2 vs. not clearly mentioned in 7 - The ecologically literate population has a high awareness of the consequences of lifestyle choices and thus has relatively low consumption and a generally low ecological footprint in 2 vs. consumer behaviour not clear in 7 - Water quality is managed through the public-private partnership in 2 vs. the roles of public and private actors not clear in 7 |
| **Megafauna, oceans, biodiversity use** | |
|  | - Protected areas limited in 2 vs extensive protected areas in 7 - Limited loss of biodiversity and modification of landscape are considered socially acceptable so long as they do not adversely affect the long term delivery of NCPs. in 2 vs. Societies have a use-orientation towards nature, but societies value and protect the self-regulating capacity of the biosphere in 7. |
| **Trade, law-rights, education, policy** | |
|  | - Strong investments in engineering education to support the world with high efficiency of the utilisation of ES in 2 vs. education is connected to conservationist and productivist legislation in 7 - High awareness of nature's contributions to nutrition, and food security in 2 vs. high awareness to regulating contributions in 7. - Trade liberalisation of food is implied in 2 with the global food system and large international cooperation in 2 vs. moderate international trade in 7 |

1. **Sharing through sparing (NN-NS or 7) vs Reciprocal stewardship (NC or 3)**

| ***SIMILARITIES*** | ***DIFFERENCES*** |
| --- | --- |
| **Economy, governance, cities, communities** | |
| - Self-determined, diverse governance - 3 - Decentralised governance in urban areas and direct surroundings in 7 | **Narrative 3**   - Most live in cities - Cities designed to cope with sustainability issues and to optimisation - People feel connected to cities than nature - Green infrastructure for leisure and recreation - Nature as virtual reality - Circular and green economy driven by technology   **Narrative 7**   - Recognition of indigenous people's and local identities and sovereignty - Shared values towards and connected to nature - Autonomous rural settlement - High tech traditional practices designed to fuel balanced human nature relationship - Resilient and functional biocultural landscape   ---   - In 7 governance is more centralised than in 3. - 7 has a stronger hierarchical distribution within larger cities, and is strongly informed by data and algorithms (while in 3 decisions are driven by social deliberation). - The economic system in 7 is more capital based than in 3. In 7 there is net economic growth while in 3 there isn’t. In 7 economic growth has been decoupled from environmental impact. - In 7 there are big cities, while in 3 these are very small (rural settlements). - In 7, there are some rural communities in the peripheries of cities, which are not attached to the place/nature, rather just to the productive sense of nature. |
| **Infrastructure, energy, transport, water** | |
| - Small scale, local scale production and consumption (e.g. energy, food) - 3 - Local and regional energy with renewables - 7 | **Narrative 3**   - Multi-modal transportation - Socio-cultural value towards freshwater as a living system   **Narrative 7**   - Nature-based solutions (in cities) with grey infrastructure (outside cities) - Clean efficient fossil free transport with global and long distance by cleantech air and hyperloops - Water, heat, energy use in circular economy framework - Zero waste, reuse materials |
| **Food, diet, agriculture, fisheries, aquaculture, land management, well-being** | |
|  | **Narrative 3**   - Wide diversity of edible species - Highly seasonal local food - Cultural value of eating - Indigenous and traditional practices - Small-scale community based management - Strong collaboration within families and communities - Strong sense of place, spiritual connections with nature - Highly heterogeneous and functional landscape - Active land sharing   **Narrative 7**   - Smart technology optimising yields using nutrient cycle, irrigation, recycle/reuse - Agriculture based on ecological principles for optimal use of ecosystem services, highly diverse and multifunctional landscape - Fresh produce from urban horticulture, plant factory and farming lead to a healthy life for urban dwellers - Protein and meat substitute at affordable costs - Sustainable and zoned fishing with monitoring - Aquaculture limited with monitoring, aims for human benefit, nutrient oriented, high use of multi-trophic systems |
| **Megafauna, oceans, biodiversity use** | |
|  | **Narrative 3**   - Biodiversity and ecosystem persistence through traditional stewardship - Conservation of culturally important species - Practical and Integrative conservation of land/sea scapes and species - Old traditions of wild species consumption is reformed   **Narrative 7**   - Wilderness and natural dynamics in protected areas - Moderately managed wilderness and protected areas - Enhanced ecological resilience through spillover effects - Cascading and teleconnected disturbance from human activities prevented - Active restoration of ecosystems - Protected area increases production of stocks outside PAs |
| **Trade, law-rights, education, policy** | |
| - Cultural fusion of ecological dynamics with community histories and priorities (3) - Ecological and productionist legislation connected to education infrastructure and social systems (7) | **Narrative 3**   - Citizens involvement, community rights, high socialisation, voluntary engagement, social networks - Gross National Happiness guides regional and international collaboration - No net economic growth - local culture based wellbeing economy - Social values than monetary values - Tech advancement reinforces interpersonal relations and cultural connectivity with nature   **Narrative 7**   - Conservationist and productivist approach - Integrated and connected through land and oceans - global security over privacy rights - IT and AI enables high tech policing |

1. **Sharing through sparing (NN-NS or 7) vs Innovative commons (NS-NC or 4)**

| ***SIMILARITIES*** | ***DIFFERENCES*** |
| --- | --- |
| **Economy, governance, cities, communities** | |
| Both have distributed (i.e., not highly centralised) governance | - Governance in 4 is more decentralised and localised with more autonomous communities In 4, settlements form a networks of well-connected medium-size to small cities and extensive rural settlements; there is no megacity - 7 is more highly urbanised, with a concentration of cities in areas that are considered ecologically/geographically appropriate for settlement. - In 7 AI and algorithms, and virtual reality play a critical role. They facilitate diverse scales of decentralisation based on the functionality of the landscapes making up and surrounding the main urban areas |
| **Infrastructure, energy, transport, water** | |
| - Low impact nature-based solutions to connect people across landscapes - Mix of green/renewable energy connected through ‘smart’ grid or collaborative systems | - While in 7, the transport infrastructure is geared toward short to medium trips, with limited global/long distance transportation, mostly by air; in 4, the transportation system is extensive across any single region and is multimodal all over (land, air, sea, public & personal, as long as it is sustainable). - In 4, most if not all consumers are also producers; only excess energy is freely traded by producing communities over the Energy Internet. - Role of markets? |
| **Food, diet, agriculture, fisheries, aquaculture, land management, well-being** | |
| - Both 7 and 4 foster innovation and sustainable intensification of the food system | - The food production system in narrative 7 is more intensive than the one in 4: fishing is strongly controlled and limited by large protected areas. - Aquaculture production is restricted to specific areas in 7, where there is also considerable use of urban space and urban gardens to produce fresh food. - By contrast, fishing as well as aquaculture production and farming are all spatially extensive in narrative 4 where ecological intensification and food production take place throughout rural and per-iurban landscapes |
| **Megafauna, oceans, biodiversity use** | |
| - Both give consideration to ecological functions | - There is a large amount of protected areas in 7, against a limited number in 4 (<14%). The focus is also different. In 7 diversity and redundancy are important to ensure resilience, in 4 protected areas are used to preserve charismatic and culturally important megafauna. - In 7, there is some interest in restoring megafauna/natural systems, including through reintroduction of species, while in 4 the primary focus is to safeguard biological and cultural heritage. - In 7, ecological functions focus primarily on natural dynamics, while the focus in 4 is on people and their social-ecological reproduction |
| **Trade, law-rights, education, policy** | |
| - Both 4 and 7 emphasise well interconnected regional trade; - Both favour a circular economy framework | - 4 is based on a regenerative social economy with principles of circularity, collaboration and commoning, while the economic system in 7 is more market and capital based - The institutional system in 7 is highly normative and top-down, and it is heavily supported by data and technology to ensure efficient regulation, control and monitoring of human activities - In 4 the laws and policies are highly deliberative and enforcement is strongly dependent upon citizen forums and community engagement. |

1. **Sharing through sparing (NN-NS or 7) vs Dynamic natures (NN-NC or 6)**

| ***SIMILARITIES*** | ***DIFFERENCES*** |
| --- | --- |
| **Economy, governance, cities, communities** | |
| - Both advocate polycentric, decentralised governance | - In 6, communities are more connected to each other and nature with values of reciprocity; - While in 7 communities concentrate in geographically suitable and ecologically feasible/selected landscapes; communities are high-tech driven, AI-based connectivity. - In 6, cities are environmentally oriented infrastructure/ buildings nature-based, locally engineered and integrated with nature, self-sufficient/reliant on locally available resources, hill-based traditional hobbit houses - In 7, cultural connections with nature are less valued, people are less connected/networked |
| **Infrastructure, energy, transport, water** | |
| - Travel by air and sea using clean energy for long distances, - Both use renewable energy with high efficiency | - In 6, infrastructure is more nature-based, locally oriented and less technology dependent - In 6, transport for short and medium distances is more inclined to adaptive, nature-based traditional and dynamic system, more personalised - In 7, for short and medium distance mass transport prefered using renewable energy and efficient technology is more prefered, less personalised - Water in 6 is treated as key to life and has legal status, while in 7, no mention of the legal status but zero waste of water, used in a closed loop (recycled) - In 6, energy is from local renewable sources and more focused on efficiency than production |
| **Food, diet, agriculture, fisheries, aquaculture, land management, well-being** | |
| - Both advocate for dynamic protected area management to enhance wilderness and protect migratory species | - In 6, land management through natural resource accounting approach, - In 7, resilience building and restoration through reintroduction of threatened species (de-extinction), - In 7, smart and technology intensive agriculture is source of food, highly engineered nutrition balanced diet, urban horticulture and fish from aquaculture are sources of food, less ecologically oriented agriculture; all aquatic areas are zoned and managed - In 6, agriculture is ecological, nature sourced, supplementing diets collected from foraged from natural ecosystems; community and eco-based fisheries management, small scale production dominated; traditional multi-trophic eco-agriculture |
| **Megafauna, oceans, biodiversity use** | |
| None | - In 7, there is high interest to restore biodiversity and species through reintroduction and de-extinction; - In 6, species are indirectly conserved as humans make way for nature and benefit from connected dynamic ecosystems |
| **Trade, law-rights, education, policy** | |
| - Both consider environmental education and education infrastructure as basis for ecological legislation and sociocultural systems | - In 6, there is a mix of ecological laws and high tech policing with citizen networks; nature protection laws and community rights; - In 7, high primacy of protectionist legislation, AI and technology supported policing, No community rights recognition; land use and tenure regulations allow for productive use of land with some limitations of negative impacts on biodiversity and climate |

**PAIRED COMPARISON BETWEEN ‘OPTIMISING NATURE’ NARRATIVE AND OTHERS**

1. **Optimising Nature (NS or 2) vs Arcology (NN or 1)**

| **SIMILARITIES** | **DIFFERENCES** |
| --- | --- |
| **Governance, cities and communities** | |
| - People live in high-tech cities - Monitoring systems which completely rely on innovative technologies. - Global top-down governance. | - Many people also live in rural areas in 2, but not in 1. |
| **Infrastructure, energy and transport** | |
| - Populations are connected by high technology communication/transportation systems. - Utilisation of clean energy. | - Human infrastructures are exclusively limited to urban areas in 1, but more dispersed and extensive, including rural areas in 2. - Focuses strongly on renewable energy from ecosystems in 2, while 1 also has focus on nuclear energy centres. |
| **Food, Diet, Agriculture, Fisheries, Aquaculture, land management, well being** | |
| - Availability of healthy and sufficient food with technology innovation. | - More diversity of food in 2 than in 1. - Extreme land-sparing in 1, but land sharing in 2. |
| **Megafauna, oceans and conservation** | |
|  | - Strong preservation of biodiversity in 1 but very limited protected area in 2. 2 also allows for biodiversity loss. |
| **Trade, law-rights, education, policy** | |
| - High education | - Education focuses on conservation in 1, but engineering on sustainable use of ecosystem services in 2. |

1. **Optimising nature (NS or 2) and Reciprocal stewardship (NS or 3)**

| ***SIMILARITIES*** | ***DIFFERENCES*** |
| --- | --- |
| **Economy, Governance, Cities, Communities** | |
| - Both have governance systems that are cognisant of nature | - 2 is highly urbanised and dense, 3 is globally distributed to rural areas - 2 is high growth and development, 3 uses National Happiness and other metrics |
| **Infrastructure, Energy, Transport, Water** | |
| - Both have energy stability | - 2 has large energy systems and distributed networks, 2 is more localised and has diverse networks - 2 has advanced public transport, 3 has diverse public transport (e.g. horse, walking). |
| **Food, Diet, Agriculture, Fisheries, Aquaculture, Land management, Well-being** | |
| - Aquaculture is used in both - Both are relatively food secure | - 2 has globalised trade, 3 has regional trade between communities - 2 has ecological extensification of agriculture but highly efficient and optimised food production, while 3 has much more agroecological systems. - 2 is dependent on food markets, 3 has greater food sovereignty |
| **Megafauna, Oceans, Biodiversity use** | |
|  | - 2 allows extinction and land expansion, 3 is strongly conservation oriented. |
| **Trade, Law-rights, Education, Policy** | |
| - Both Narratives have recognition of women’s contribution to the economy and knowledge | - 2 follows a Green Economy growth narrative, 3 is more solidarity economics oriented, with no net economic growth |

1. **Optimising nature (NS or 2) and Innovative commons (NS or 4)**

| **SIMILARITIES** | **DIFFERENCES** |
| --- | --- |
| **Governance, cities and communities** | |
| - Networks of communities, cities and rural settlements that are well-connected. | - 2 has top-down centralised governance, with flexibility for adapting to city and rural settings, while in 4, governance is a decentralised and polycentric - In 2, cities are extensive, big and designed to utilise ecosystem services efficiently but 4 does not have mega-cities and the size varies |
| **Infrastructure, energy and transport** | |
| - Based on renewable energy, well-connected ‘smart’ grid - Transport is multimodal: sea, air, river and land - Transportation infrastructure aims to reduce impacts on the environment for connecting people | - In 4 energy trade can happen over the internet and in 2 energy production is controlled by top-down regulation and coordination - In 4, transportation infrastructure aims to reduce impact on all aspects of the environment while in 2 it focuses on minimising impacts on ecological functions only. |
| **Food, Diet, Agriculture, Fisheries, Aquaculture, land management, well being** | |
| - Both 2 and 4 foster innovation to improve productivity and sustainability of food systems. - Eco-agriculture is dominant in mosaic landscapes. | - In 4, the production system is through nature-based innovations and ecological intensification while in 2 it is based on ecological extensification and optimising productivity through innovative technology in general. - In 2, fisheries is large-scale and while in 4 it is small-scale |
| **Megafauna, oceans and conservation** | |
| - Limited protected areas | - More protected areas (<14%) in 4 than in 2. - In 2, conservation focuses on maintenance of ecosystem functions while in 4 protected areas are designed to safeguard biological and cultural heritage |
| **Trade, law-rights, education, policy** | |
|  | - In 2, law is top-down and adapted for cities and rural setting - In 4 citizen forums are key to enforcement while law emphasises community rights and local engagement. |

1. **Optimising Nature (NS or 2) vs Dynamic natures (NS or 6)**

| **SIMILARITIES** | **DIFFERENCES** |
| --- | --- |
| **Governance, cities and communities** | |
|  | - In 6, governance is polycentric with autonomy communities, while in 2 it is top-down centralised governance with flexibility for adapting to city and rural settings. - In 6, cities are dynamic, flexible and adapted to nature through movement e.g., ‘nomadic cities, while in 2 cities are extensive and designed to utilise ecosystem services efficiently. - In 6, community-driven demand for local control over resources, connecting to the value of recipicality, harmony and relationality while in 2, communities can choose their life-style and enjoy the benefits from nature while they are strictly regulated by the government to ensure the functionality of ecosystems. |
| **Infrastructure, energy and transport** | |
| - Strong relevance and development of green/renewable energy - Use new technology to use natural energy sources such as wind, tide, geothermal. | - In 6, renewable energy development heavily considers and is based on traditional knowledge while in 2, renewable energy is solely developed through innovative engineering. - In 6 focus on self-sufficiency and energy conservation while in 2 it is well-connected by smart grid to increase energy use efficiency. - In 6, travel is dominated by air and sea because of the limited road system, while in 2 cities and rural areas worldwide are well-connected by all means of transportation. |
| **Food, Diet, Agriculture, Fisheries, Aquaculture, land management, well being** | |
| - Use of a multi-trophic aquaculture system. | - In 2, food production is maximised through ecological extensification - almost all productive ecosystems are utilised for food production with optimal management to conserve ecological functions/biodiversity to ensure sustainable food production while in 6, harvest relies on traditional system that have evolved and adapted to ecological dynamics, including agroforestry and permaculture are linked to traditional technology. - In 6 Fishing is limited to small-scale fisheries for local communities and livelihood while in 2 it is large-scale fisheries that are aiming for yield and efficiency. - In 6, Aquaculture focuses on mixed aquatic-land farms while in 2, all systems are developed as long as they enhance productivity. |
| **Megafauna, oceans and conservation** | |
|  | - In 6 there is a dynamic protected landscape but in 2 it has limited protected areas; - 6 recognizes community and traditional rights while in 2 conservation measures are top-down controlled. |
| **Trade, law-rights, education, policy** | |
| - High education and strong environmental education. | - In 2, a globalised, highly integrated economy while in 6, it focuses on the local economy. - In 2 there is a strong focus on engineering with ecology knowledge to support ecosystem services extraction while in 6 it has strong environmental education that is based on traditional and cultural background. |

1. **Optimising Nature (NS or 2) vs Sharing through sparing (NS or 7)**

| **SIMILARITIES** | **DIFFERENCES** |
| --- | --- |
| **Governance, cities and communities** | |
| - Governance is informed by knowledge to ensure efficiency, not community focused | - In 2, it is highly top-down centralised governance while in 7, governance is decentralised to scale determined by main urban area and its surrounding agriculture/wilderness (2 is more centralised than 7) |
| **Infrastructure, energy and transport** | |
| - Green/renewable energy connected with a ‘smart’ grid. - Both avoid impacts on ecosystem functions and dynamics that are needed for benefits to people | - In 7, stronger focus on conservation of natural systems while in 2, stronger focus on maintenance ecological functions to support ecosystem services. - In 7 transportation/infrastructure recognize natural dynamics and are designed around that while in 2 transportation/infrastructure are extensively designed through engineering solutions. |
| **Food, diet, agriculture, fisheries, aquaculture, land management, well-being** | |
| - Very efficient and sustainable food production system | - In 7, fisheries are limited by large protected areas while in 2 fisheries happen in all productive areas. - In 7, because of the need to allocate more space for nature, it needs much limitation in the diets to fit within nature’s capacity while in 2, people can make their choices of food and lifestyle more freely because of more extensive use of nature. - More intensive food production system in 7 than in 2. |
| **Megafauna, oceans and conservation** | |
| - Focus on conservation of ecological functions | - Large protected areas in 7 but limited protected areas in 2. - Some interest in restoring megafauna/natural systems e.g., through reintroduction of species in 7 while in 2 it focuses on maintenance of ecological functions, even at the expense of losing some species if needed. - In 7, ecological functions focus on natural dynamics (with assumption that it would benefit people) while in 2, ecological functions focus strongly on direct/indirect benefits to people. |
| **Trade, law-rights, education, policy** | |
| - Trade is well interconnected - Top-down and heavily supported by data and technology to ensure efficient regulation, control and monitoring of human activities. | - In 7, it is more focused on regional trade because of the larger protected areas while in 2 it is globalised to ensure efficient use and flows of ecosystem services. |

**PAIRED COMPARISON BETWEEN ‘INNOVATIVE COMMONS’ NARRATIVE AND OTHERS**

1. **Innovative commons (NS-NC or 4) vs Arcology (NN or 1)**

| ***SIMILARITIES*** | ***DIFFERENCES*** |
| --- | --- |
| **Economy, governance, cities, communities** | |
|  | - 1 has top-down UN-Style centralised governance, while in 4, governance is a decentralised and polycentric - 1 is based on megacities and arcologies while 4 is based on a networks of well-connected medium-size to small cities and extensive rural settlements |
| **Infrastructure, energy, transport, water** | |
|  | - 4 is based on renewable energy, well-connected ‘smart’ grid, while 1 is powered clean nuclear energy t - There is an extensive transportation system In 4, while in 1 there is no extensive transportation infrastructure out of the cities |
| **Food, diet, agriculture, fisheries, aquaculture, land management, well-being** | |
| - Both 1 and 4 foster innovation | - In 1 lab-produced synthetic food predominate, against food produced and collected in largely rural landscapes in 4 - In 1, fishing is very limited and controlled, while in 4 it is fairly extensive |
| **Megafauna, oceans, biodiversity use** | |
|  | - Most of the planet (70%) is PA in 1, while in 4 it is <14%) - In 1, the fundamental aim is to preserve wild nature in pristine state, while in 4 protected areas are primarily designed to safeguard biological and cultural heritage |
| **Trade, law-rights, education, policy** | |
|  | - In 1, laws are highly policed, normative and top-down to enforce strong restrictions on families and access to the wild. - In 4 the laws and policies are highly deliberative and enforcement is heavily dependent upon citizen forums and community engagement. |

1. **Innovative commons (NS-NC or 4) vs Optimising nature (NS or 2)**

| ***SIMILARITIES*** | ***DIFFERENCES*** |
| --- | --- |
| **Economy, governance, cities, communities** | |
| - Networks of communities, cities and rural settlements that are well-connected. | - 2 has top-down centralised governance, with flexibility for adapting to city and rural settings, while in 4, governance is a decentralised and polycentric - In 2, cities are extensive, big and designed to utilise ecosystem services efficiently but 4 does not have mega-cities and the size varies |
| **Infrastructure, energy, transport, water** | |
| - Based on renewable energy, well-connected ‘smart’ grid - Transport is multimodal: sea, air, river and land - Transportation infrastructure aims to reduce impacts on the environment for connecting people and goods | - In 4 energy trade can happen over the internet and in 2 energy production is controlled by top-down regulation and coordination - In 4, transportation infrastructure aims to reduce impact on all aspects of the environment while in 2 it focuses on minimising impacts on ecological functions only. |
| **Food, diet, agriculture, fisheries, aquaculture, land management, well-being** | |
| - Both 2 and 4 foster innovation to improve productivity and sustainability of food systems. - Eco-agriculture is dominant in mosaic landscapes. | - In 4, the production system is through nature-based innovations and ecological intensification while in 2 it is based on ecological extensification and optimising productivity through innovative technology in general. - In 2, fisheries is large-scale and while in 4 it is small-scale |
| **Megafauna, oceans, biodiversity use** | |
| - Limited protected areas | - More protected areas (<14%) in 4 than in 2. - In 2, conservation focuses on maintenance of ecosystem functions while in 4 protected areas are designed to safeguard biological and cultural heritage |
| **Trade, law-rights, education, policy** | |
|  | - In 2, law is top-down and adapted for cities and rural setting - In 4 citizen forums are key to enforcement while law emphasises community rights and local engagement. |

1. **Innovative commons (NS-NC or 4) vs Reciprocal stewardship (NC or 3)**

| ***SIMILARITIES*** | ***DIFFERENCES*** |
| --- | --- |
| **Economy, governance, cities, communities** | |
| - Both have decentralised systems of governance and power. - The democratic nature of collaboration, social deliberation is shared by both narratives and is very frequent at different scales. | - In 4 governance is polycentric and decentralised (globally and locally). This means there exists some level of hierarchical coordination between multiple nodes (i.e. regions, countries, settlements). However, in 3 governance is very horizontal between autonomous entities (communities and settlements); almost a comunist anarchy. [Obs. A node include multiple entities] - In 3 the cities are smaller than in 4. - The landscape in 3 is a network of autonomous entities, while in 4 the networks include nodes of embedded entities. |
| **Infrastructure, energy, transport, water** | |
| - Both have novel infrastructure incorporating indigenous and local materials and knowledge. - Their energy systems are similar; both have highly decentralised, autonomous units of renewable energy and barter trade of energy over the internet of things is possible. - Their transport infrastructure is similar; both are based on innovation oriented towards low environmental impact and community connectivity. - Both 3 and 4 can have small dams stemming from local traditions and adapted to the ecology of the landscape. | - While in 3 advanced technology is specialised to foster social cohesion and personal fulfilment through fusion with nature, in 4 it is oriented to optimise productive functions and social well-being. - In 4 transport is less multimodal than in 3, since in 4 the transport system has been more homogenised. In 3 inter-settlements transportation is less frequent because settlements are more autonomous. - 4 can have bigger dams than 3. |
| **Food, diet, agriculture, fisheries, aquaculture, land management, well-being** | |
| - Both visions have productive systems that include nature-based innovations (e.g. mycorrhizal symbioses, bacterias). - Agroecological practices are common to both systems. - Landsharing is common to both systems. | - In 4 monocultures are rare to inexistent, while in 3 there are nonexistent. - In 3 agriculture and diet are more seasonal than in 4. |
| **Megafauna, oceans, biodiversity use** | |
|  | - In 3 conservation is through co-management of land (no PAs), while in 4 there are some PAs (no more than 14%). - In 3 biodiversity rooted in relational values; while in 4 aimed to safeguard biological and cultural heritage |
| **Trade, law-rights, education, policy** | |
| - Community-based “exchange”. | - In 3 no net economic growth. In 4 economic growth but decoupled from environmental impact. - Monetary exchange in 3 is very limited, while in 4 money is more socially valued. |

1. **Innovative commons (NS-NC or 4) vs Dynamic natures (NN-NC or 6)**

| ***SIMILARITIES*** | ***DIFFERENCES*** |
| --- | --- |
| **Economy, governance, cities, communities** | |
| - Governance is polycentric with community autonomy in both 4 and 6 - Global governance is decentralised in 4 and 6 - No megacities in both 4 and 6 - Both 4 and 6 are based on the power of communities; there is community-driven exercise of local control over resources - Laws emphasise community rights, and citizens engagement in both 4 and 6 | - In 4, global governance is decentralised to the regions while in 6 it is decentralised to quasi autartic local entities. - 4 is more globalised than 6, which is more local. - In 6, cities are dynamic, flexible and adapted to nature through movement e.g., ‘nomadic cities, while in 4 cities and rural settlements are fixed and extensively connected throughout the landscape. - The system of community representation is more horizontal in 6 and more networked in 4 (more with multi-level authority). - In 6, direct interconnectivity of communities is high, while in 4, networks are stronger and connectivity with trade is higher. |
| **Infrastructure, energy, transport, water** | |
| - Both 4 and 6 are based on community and traditional rights |  |
| **Food, diet, agriculture, fisheries, aquaculture, land management, well-being** | |
|  |  |
| **Megafauna, oceans, biodiversity use** | |
| - Both 4 and 6 are based on community and traditional rights | - Dynamic protected landscapes mark out narrative 6 where humans increasingly make way for nature; - By contrast, in 4 the amount of protected areas is limited (no more than 14%) while landscapes are widely invested by communities through social-ecological approaches. |
| **Trade, law-rights, education, policy** | |
| - Both are based on a regenerative   social economy with principles of circularity, collaboration and commoning.   - Strong environmental education and awareness (Although more autarchic, 6 does not prevent the exchange of knowledge across long distances). | - In 4, trade is more extensive including trade and sharing over the Internet. |

1. **Innovative commons (NS-NC or 4) vs Sharing through sparing (NN-NS or 7)**

| ***SIMILARITIES*** | ***DIFFERENCES*** |
| --- | --- |
| **Economy, governance, cities, communities** | |
| - Both have distributed (i.e., not highly centralised) governance | - Governance in 4 is more decentralised and localised with more autonomous communities In 4, settlements form a networks of well-connected medium-size to small cities and extensive rural settlements; there is no megacity - In 4, global governance is decentralised to more or less equal regions - 7 is more highly urbanised, with a concentration of cities in areas that are considered ecologically/geographically appropriate for settlement - In 7 AI and algorithms, and virtual reality play a critical role. They facilitate diverse scales of decentralisation based on the functionality of the landscapes making up and surrounding the main urban areas - People’s sense of place has largely disappeared in 7, while cultural connections with nature are not highly valued. By contrast, people have a strong sense of place, culture and identity in 4 |
| **Infrastructure, energy, transport, water** | |
| - Low impact nature-based solutions to connect people across landscapes - Mix of green/renewable energy connected through ‘smart’ grid or collaborative systems | - While in 7, the transport infrastructure is geared toward short to medium trips, with limited global/long distance transportation, mostly by air; in 4, the transportation system is extensive across any single region and is multimodal all over (land, air, sea, public & personal, as long as it is sustainable). - The energy system in 7 is more unified and centrally-coordinated than in 4. The smart microgrids are operated to enable coordinated shifting of energy supply in response to the fluctuation of demand across self-sufficient cities and urban landscapes. - By contrast, the energy system in 4 is based on autonomous energy producing and consuming communities and the free trade of excess energy over the Energy Internet (EI). - In 7, energy producers and consumers form separate poles and the microgrids regulate the whole to make it self-sufficient; in 4, most if not all consumers are also producers; only excess energy is freely traded by producing communities over the Energy Internet. Market-based supply and demand pricing is predominant in 7, while in 4, collaborative commoning, reciprocal credit and barter trade are more essential to trade over the EI. |
| **Food, diet, agriculture, fisheries, aquaculture, land management, well-being** | |
| - Both 7 and 4 foster innovation and sustainable intensification of the food system | - The food production system in narrative 7 is more intensive than the one in 4: fishing is strongly controlled and limited by large protected areas. Aquaculture production is restricted to specific areas in 7, where there is also considerable use of urban space and urban gardens to produce fresh food. - By contrast, fishing as well as aquaculture production and farming are all spatially extensive in narrative 4 where ecological intensification and food production take place throughout rural and peri-urban landscapes |
| **Megafauna, oceans, biodiversity use** | |
| - Both have protected areas - Both give consideration to ecological functions | - There is a large amount of protected areas in 7, against a limited number in 4 (<14%) - In 7, there is some interest in restoring megafauna and natural systems, including through reintroduction of species, while in 4 the primary focus is to safeguard biological and cultural heritage. - In 7, ecological functions focus primarily on natural dynamics, while the focus in 4 is on people and their social-ecological reproduction |
| **Trade, law-rights, education, policy** | |
| - Both 4 and 7 emphasise well interconnected regional trade; - Both favour a circular economy framework | - 4 is based on a regenerative social economy with principles of circularity, collaboration and commoning, while the economic system in 7 is more market and capital based - The institutional system in 7 is highly normative and top-down, and it is heavily supported by data and technology to ensure efficient regulation, control and monitoring of human activities - In 4 the laws and policies are highly deliberative and enforcement is strongly dependent upon citizen forums and community engagement. |

**PAIRED COMPARISON BETWEEN ‘RECIPROCAL STEWARDSHIP’ NARRATIVE AND OTHERS**

1. **Reciprocal stewardship (NC or 3) vs Arcology (NN or 1)**

| ***SIMILARITIES*** | ***DIFFERENCES*** |
| --- | --- |
| **Economy, governance, cities, communities** | |
|  | - In 1 governance is strongly top-down (very centralised), while in 3 it is strongly bottom-up and horizontal. - In 1 there are no rural areas, at all, while in 3 landscapes are made up of rural patchy areas. - In 1 everyone lives in cities, while in 3 the majority of people live in rural areas. - In 1 families are really small, they maybe even disappeared and therefore the relationship within “urban communities” is very horizontal. In 3, families are big and very interconnected within rural settlements. |
| **Infrastructure, energy, transport, water** | |
| - Both 3 and 1 use innovative energy systems. | - In 1 there is no infrastructure outside cities, while in 3 infrastructure is integrated throughout the landscape. - In 1 energy is not renewable (nuclear) and highly centralised, while in 3 completely renewable and decentralised. - In 1 transport systems are focused on speed, efficiency, trade and mobility. In 3 transport systems focus on social connectivity. - In 3 transport systems are highly multimodal, while in 1 there are not. - In 1 water systems are closed and use innovative systems (i.e. microbiological processes) for sanitising. In 1 hydrological systems are “wild” and not intervened. In 3 hydrological systems are intervened throughout the landscape, but at watershed level with small level extractions. |
| **Food, diet, agriculture, fisheries, aquaculture, land management, well-being** | |
|  | - In 1 agricultural diversity is much lower than in 3, and fresh food is very limited (i.e. produced in labs and house roofs). - In 1 food production isn't seasonal, while in 3 is highly seasonal. - In 1 diet is driven by technical and resource capacity, while in 3 is driven by cultural heritage and value. - In 1 fishing and aquaculture are restricted to highly controlled MPAs, while in 3 fisheries are small-scale and community-based managed throughout the seascape. - In 1 land-sparing is maximised, while in 3 land-sharing is maximised. - In 1, well-being is primarily generated through virtual reality and supported AI, while in 3 is driven by the strong spiritual and mental connection with nature supported by the constant practice into it. |
| **Megafauna, oceans, biodiversity use** | |
|  | - In 1 there are mega PAs, untouched natures, while in 3 there aren’t PAs and biodiversity protection is achieved by the co-management of the landscape. A similar protection system is applied to oceans. - While intrinsic value of nature dominantes in 1, there is some instrumental use of biodiversity that is limited to engineered systems (i.e. hydroponic productivity of vegetables in labs, and microbiological treatment of waste). However, in 3, the use of biodiversity stems from a relational value and reciprocal relationship. |
| **Trade, law-rights, education, policy** | |
| - In both visions there is trade of knowledge and services. - In both visions there is an anarchical system of power, in which people (in 1) and communities (in 3) are self organised. | - In 3 the trade of goods is more frequent than in 1, but it focuses on the social value of things rather than their monetary value. - In 3 laws emphasise community rights and socialisation is high. However, in 1 law focus on the optimisation and operationalization of the metropolis. - In 3 laws stem from relational and cultural fulfilment, while in 1 stem from ecological efficiency. - In 3 education emphasises the interdependence between communities-people and nature, while in 1 social organisation within cities. |

1. **Reciprocal stewardship (NC or 3) vs Optimising nature (NS or 2)**

| ***SIMILARITIES*** | ***DIFFERENCES*** |
| --- | --- |
| **Economy, governance, cities, communities** | |
| - Both have governance systems that are cognisant of nature | - 2 is highly urbanised and dense, 3 is globally distributed to rural areas - 2 there is economic growth decoupled from environmental impacts, while in 3 there is not economic growth. - In 2 governance remains top-down with nations or states enacting regulations, while in 3 governance is highly bottom-up being settlements that define their rules. - In 2 communities are rather networks, which are driven by an interest in exchanging knowledge or commodities. In contrast, in 3, communities are culturally embedded, driven by a sense of tradition and family cooperation. |
| **Infrastructure, energy, transport, water** | |
| - Both have energy stability | - 2 has large energy systems and distributed networks, 2 is more localised and has diverse networks - 2 has advanced public transport, 3 has diverse public transport (e.g. horse, walking). |
| **Food, diet, agriculture, fisheries, aquaculture, land management, well-being** | |
| - Aquaculture is used in both - Both are relatively food secure | - 2 has globalised trade, 3 has regional trade between communities - 2 has intensive farming systems, 3 has much more agroecological systems. - 2 is dependent on food markets, 3 has greater food sovereignty. - In 2 the instrumental value of nature supports people’s well-being, while in 3 is relational value. In 2 the use of nature is maximised to satisfy a wide range of needs, while in 3 the use of nature to meet people needs accounts for existing trade-offs and is, therefore, less intense. |
| **Megafauna, oceans, biodiversity use** | |
|  | - 2 allows extinction and land expansion, 3 is strongly conservation oriented. |
| **Trade, law-rights, education, policy** | |
| - Both Narratives have recognition of women’s contribution to the economy and knowledge | - 2 follows a Green Economy growth narrative, 3 is more solidarity economics oriented, with no net economic growth |

1. **Reciprocal stewardship (NC or 3) vs Innovative commons (NS-NC or 4)**

| ***SIMILARITIES*** | ***DIFFERENCES*** |
| --- | --- |
| **Economy, governance,cities, communities** | |
| - Both have decentralised systems of governance and power. - The democratic nature of collaboration, social deliberation is shared by both narratives and is very frequent at different scales. | - In 4 governance is polycentric and decentralised (globally and locally). This means there exists some level of hierarchical coordination between multiple nodes (i.e. regions, countries, settlements). However, in 3 governance is very horizontal between autonomous entities (communities and settlements); almost a comunist anarchy. [Obs. A node include multiple entities] - In 3 the cities are smaller than in 4. - The landscape in 3 is a network of autonomous entities, while in 4 the networks include nodes of embedded entities. |
| **Infrastructure, energy, transport, water** | |
| - Both have novel infrastructure incorporating indigenous and local materials and knowledge. - Their energy systems are similar; both have highly decentralised, autonomous units of renewable energy and barter trade of energy over the internet of things is possible. - Their transport infrastructure is similar; both are based on innovation oriented towards low environmental impact and community connectivity. - Both 3 and 4 can have small dams stemming from local traditions and adapted to the ecology of the landscape. | - While in 3 advanced technology is specialised to foster social cohesion and personal fulfilment through fusion with nature, in 4 it is oriented to optimise productive functions and social well-being. - In 4 transport is less multimodal than in 3, since in 4 the transport system has been more homogenised. In 3 inter-settlements transportation is less frequent because settlements are more autonomous. - 4 can have bigger dams than 3. |
| **Food, diet, agriculture, fisheries, aquaculture, land management, well-being** | |
| - Both visions have productive systems that include nature-based innovations (i.e. mycorrhizal symbioses, bacterias). - Agroecological practices are common to both systems. - Land-sharing is common to both systems. | - In 4 monocultures are rare to inexistent, while in 3 there are nonexistent. - In 3 agriculture and diet is more seasonal than in 4. |
| **Megafauna, oceans, biodiversity use** | |
|  | - In 3 conservation is through co-management of land (no PAs), while in 4 there are some PAs (no more than 14%). |
| **Trade, law-rights, education, policy** | |
| - Community-based “exchange”. | - In 3 no net economic growth. In 4 economic growth but decoupled from environmental impact. - Monetary exchange in 3 is very limited, while in 4 money is more socially valued. |

1. **Reciprocal stewardship (NC or 3) vs Dynamic natures (NN-NC or 6)**

| ***SIMILARITIES*** | ***DIFFERENCES*** |
| --- | --- |
| **Economy, governance, cities, communities** | |
| - In both 3 and 6 governance systems are polycentric with community autonomy - Global governance is decentralised in 3 and 6. - No megacities in both 3 and 6. - Both 3 and 6 are based on the power of communities; there is community-driven exercise of local control over resources. - Laws emphasise community rights, and citizens engagement in both 3 and 6. - The system of community representation is highly horizontal in both 3 and 6. | - In 3 governance systems are very diverse based on local and cultural specificities. However, in 6 governance systems are less diverse than in 3, since they need to be more flexible and adaptable to nature dynamics - In 6, cities are dynamic, flexible and adapted to nature through movement (e.g. nomadic cities), while in 3 cities are rare and very small (rural settlements), and are fixed and extensively connected throughout the landscape. - In 3 governance systems are more driven by indigenous and local communities' knowledge systems and rights over their lands, than in 6. In 6 there isn’t much of a level of attachment to land since they are mostly nomadic. |
| **Infrastructure, energy, transport, water** | |
| - Both 3 and 6 are based on decentralised and autonomous energy systems. - Both rely on nature-based innovation and indigenous local knowledge. - Energy developments are based in both cases on renewables - energy, solar, wind, tide, geothermal, etc.-, with high levels of community sovereignty. | - In 3, energy systems are more decentralised than in 6, since communities are highly autonomous (fixed) entities. - In 3, energy trade can happen over the Internet of Things, not in 6 where society is more focused on saving energy for total self-sufficiency. - In 6, travel is dominated by air and sea because of a limited road system, while in 3 rural areas are more connected by diverse means of transportation. In 3, transportation infrastructure is built to reduce impact on the environment and foster social connectivity. - In 6, energy systems are more diversified and eclectic than in 3. In 3 energy innovations are highly selected for those features that enhance social connectivity and well-being. |
| **Food, diet, agriculture, fisheries, aquaculture, land management, well-being** | |
| - Both narratives rely heavily on community-based systems and smallholder operated systems for food production. - Fishing is mostly or uniquely small-scale for local communities and livelihood; it is very coastal and continental. - Aquaculture develops in similar ways in agro-ecological systems . | - Production in 6 is more intensive and more seasonal than in 3. Diets in 6 can include synthesised food, as long as it can effectively help spare nature. - In 3 fisheries can use larger areas than in 6 where MPAs are more numerous and cover a greater area (in 3 there are no MPAs since the seascape is co-managed for conservation and production). |
| **Megafauna, oceans, biodiversity use** | |
| - Both 3 and 6 are based on community and traditional rights. | - Dynamic protected landscapes mark out narrative 6 where humans increasingly make way and space for nature; - By contrast, in 3 there are no protected areas; protection of biodiversity happens uniquely through co-managing of the scape, focusing on culturally relevant species. |
| **Trade, law-rights, education, policy** | |
| - Both systems are community-based and self-reliant and apply circular economic principles. - Strong environmental education and awareness. Although more autarchic, 6 does not prevent the exchange of knowledge across long distances. | - In 3, trade is important and extensive among neighbours over some distance, while in 6 each community seeks complete self-reliance. - Trade exchange in 6 is heavily based on the use of local currencies. In 6 monetary exchange is more important than barter trade, compared to 3 where it predominates. |

1. **Reciprocal stewardship (NC or 3) vs Sharing through sparing (NN-NS or 7)**

| ***SIMILARITIES*** | ***DIFFERENCES*** |
| --- | --- |
| **Economy, governance, cities, communities** | |
|  | - In 7 governance is more centralised than in 3. 7 has a stronger hierarchical distribution within larger cities, and is strongly informed by data and algorithms (while in 3 decisions are driven by social deliberation). - The economic system in 7 is more capital based than in 3. In 7 there is net economic growth while in 3 there isn’t. In 7 economic growth has been decoupled from environmental impact. - In 7 there are big cities, while in 3 these are very small (rural settlements). - In 7, there are some rural communities in the peripheries of cities, which are not attached to the place/nature, rather just to the productive sense of nature. |
| **Infrastructure, energy, transport, water** | |
| - Both have decentralised energy systems. However, in 3 it’s focused on the local level, in 7 at the city-region level. | - In 7 infrastructure is much larger than in 3, and it concentrates within cities and productive landscapes. - In 7, the transport system is very homogeneous (less multimodal than 3), and focuses on speed and functionality. In 3 transport systems are highly multimodal and focus on connectivity rather than speed. - In 7 there are much larger dams than in 3. However these still permit the natural flow of freshwater systems. |
| **Food, diet, agriculture, fisheries, aquaculture, land management, well-being** | |
|  | - In 7 there are monocrops concentrated within a buffer around cities, while in 3 there are no monocrops. - In 7 strong land sparing and in 3 strong land sharing. - In 7 well-being is supported by virtual reality and green areas within cities, although some minimal interaction with nature happens within productive buffers around cities. In 3 well-being is strongly driven by constant and embedded interactions with nature; spiritual and mental. |
| **Megafauna, oceans, biodiversity use** | |
|  | - In 3 conservation is through co-management of land (no PAs), while in 7 there are huge areas of protected areas. In 3, the relational and cultural connection with certain species/communities determines whether they exist or not, while in 7 large areas are protected including all life. |
| **Trade, law-rights, education, policy** | |
|  | - In 3 trade/exchange is mostly local and small scale. In 7 there is some global trade, but much happens between and within urban regions and their surrounding production landscapes. |

**PAIRED COMPARISON BETWEEN ‘DYNAMIC NATURES’ NARRATIVE AND OTHERS**

1. **Dynamic Natures (NN-NC or 6) vs Arcology (NN or 1)**

| ***SIMILARITIES*** | ***DIFFERENCES*** |
| --- | --- |
| **Economy, governance, cities, communities** | |
| - High education and strong environmental education. | - Top-down governance vs Decentralised - In 6, governance is polycentric with autonomy communities, while in 1 it is top-down centralised governance - In 1, people live in high-tech cities while in 6, cities are dynamic, flexible and adapted to nature ‘nomadic cities. - In 6, community-driven demand for local control over resources, connecting to the value of recipicality, harmony and relationality while in 1, communities are strictly urban. |
| **Infrastructure, energy, transport, water** | |
| - Green infrastructure - Self sufficiency - Free river flowing | - In 1 nuclear centralised clean energy and in 6 renewable, autonomous systems. - There is no infra-structure outside cities in 1, while in 6 we find dynamic housing embedded (some nomadic). - Underground hyperloops and drones are used to connect cities to minimise anthropogenic impacts on nature in 1 and in 6, dynamic transport that uses tides, wind power and new technology that really is able to capture these natural forces, building on traditional knowledge (e.g. Polynesian/pacific island boats). Travel by air and sea is enhanced. |
| **Food, diet, agriculture, fisheries, aquaculture, land management, well-being** | |
| - Use of aquatic ecosystems to food resources | - In 1, lab food development (no fresh food) while in 6, harvest relies on traditional systems that have evolved and adapted to ecological dynamics (agroforestry, permaculture, traditional technology). - In 6 fishing small-scale for local communities and livelihoods while in 1 very limited catch because of MPAs. - In 6, aquaculture focuses on mixed aquatic-land farms, however in 1 the production is restricted to specific areas and dominated by seaweed and shellfish in scale that are well-within ecological capacity. |
| **Megafauna, oceans, biodiversity use** | |
| - Connectivity - No take MPA - Aquaculture based on local species in 1 and local consumption in 6 | - In 6 Dynamic protected landscape but in 1 all protected; - In 6 recognize community and traditional rights while in 1 conservation measures are top-down controlled (highly connected 'pristine' areas, maximised for nature) - Maximise production through multitrophic in 1 but no large production in 6 - co-managed government fishing in 6 |
| **Trade, law-rights, education, policy** | |
| - High education and strong environmental education. - Laws and regulations are based on ecology | - In 1, a globalised, highly integrated economy while in 6, it focuses on the local economy. - In 1, the high-tech economy is based on services, digital economy, exchange of goods between cities possible with novel technologies, economy of knowledge in 6 it has strong environmental education that is based on traditional and cultural background. |

1. **Dynamic natures (NN-NC or 6) vs Optimising Nature (NS or 2)**

| **SIMILARITIES** | **DIFFERENCES** |
| --- | --- |
| **Governance, cities and communities** | |
|  | - In 6, governance is polycentric with autonomy communities, while in 2 it is top-down centralised governance with flexibility for adapting to city and rural settings. - In 6, cities are dynamic, flexible and adapted to nature through movement e.g., ‘nomadic cities, while in 2 cities are extensive and designed to utilise ecosystem services efficiently. - In 6, community-driven demand for local control over resources, connecting to the value of recipicality, harmony and relationality while in 2, communities can choose their life-style and enjoy the benefits from nature while they are strictly regulated by the government to ensure the functionality of ecosystems. |
| **Infrastructure, energy and transport** | |
| - Strong relevance and development of green/renewable energy - Use new technology to use natural energy sources such as wind, tide, geothermal. | - In 6, renewable energy development heavily considers and is based on traditional knowledge while in 2, renewable energy is solely developed through innovative engineering. - In 6 focus on self-sufficiency and energy conservation while in 2 it is well-connected by smart grid to increase energy use efficiency. - In 6, travel is dominated by air and sea because of the limited road system, while in 2 cities and rural areas worldwide are well-connected by all means of transportation. |
| **Food, Diet, Agriculture, Fisheries, Aquaculture, land management, well being** | |
| - Use of a multi-trophic aquaculture system. | - In 2, food production is maximised through ecological extensification - almost all productive ecosystems are utilised for food production with optimal management to conserve ecological functions/biodiversity to ensure sustainable food production while in 6, harvest relies on traditional system that have evolved and adapted to ecological dynamics, including agroforestry and permaculture are linked to traditional technology. - In 6 Fishing is limited to small-scale fisheries for local communities and livelihood while in 2 it is large-scale fisheries that are aiming for yield and efficiency. - In 6, Aquaculture focuses on mixed aquatic-land farms while in 2, all systems are developed as long as they enhance productivity. |
| **Megafauna, oceans and conservation** | |
|  | - In 6 Dynamic protected landscape but in 2 it has limited protected areas; - In 6 Recognize community and traditional rights while in 2 conservation measures are top-down controlled. |
| **Trade, law-rights, education, policy** | |
| - High education and strong environmental education. | - In 2, a globalised, highly integrated economy while in 6, it focuses on the local economy. - In 2Strong focus on engineering with ecology knowledge to support ecosystem services extraction while in 6 it has strong environmental education that is based on traditional and cultural background. |

1. **Dynamic Natures *(*(NN-NC or 6) vs Reciprocal stewardship (NC or 3)**

| ***SIMILARITIES*** | ***DIFFERENCES*** |
| --- | --- |
| **Economy, governance, cities, communities** | |
| - In both 3 and 6 governance systems are polycentric with community autonomy - Global governance is decentralised in 3 and 6. - No megacities in both 3 and 6. - Both 3 and 6 are based on the power of communities; there is community-driven exercise of local control over resources - Laws emphasise community rights, and citizens' engagement in both 3 and 6. - The system of community representation is highly horizontal in both 3 and 6. | - In 3 governance systems are very diverse based on local and cultural specificities. However, in 6 governance systems are less diverse than in 3, since they need to be more flexible and adaptable to nature dynamics - In 6, cities are dynamic, flexible and adapted to nature through movement e.g., ‘nomadic cities, while in 3 cities are rare and very small (rural settlements), and are fixed and extensively connected throughout the landscape. - In 3 governance systems are more driven by indigenous and local communities' knowledge systems and rights over their lands, than in 6. In 6 there isn’t much of a level of attachment to land since they are mostly nomadic. |
| **Infrastructure, energy, transport, water** | |
| - Both 3 and 6 are based on decentralised and autonomous energy systems. - Both rely on nature-based innovation and indigenous local knowledge. - Energy developments are based in both cases on renewables - energy, solar, wind, tide, geothermal, etc.-, with high levels of community sovereignty | - In 3 the energy systems are more fragmented than in 6, since communities are highly autonomous (fixed) entities. - In 3, energy trade can happen over the Internet of Things, not in 6 where society is more focused on saving energy for total self-sufficiency. - In 6, travel is dominated by air and sea because of the limited road system, while in 3 rural areas are more connected by diverse means of transportation. In 3, transportation infrastructure is built to reduce impact on the environment and foster social connectivity. - In 6, energy systems are more diversified and eclectic than in 3. In 3 energy innovations are highly selected for those features that enhance social connectivity and well-being. |
| **Food, diet, agriculture, fisheries, aquaculture, land management, well-being** | |
| - Both narratives rely heavily on community-based systems and smallholder operated systems for food production. - Fishing is mostly or uniquely small-scale for local communities and livelihood; it is very coastal and continental. - Aquaculture develops in similar ways in agro-ecological systems. | - Production in 6 is more intensive and more seasonal than in 3, while the diets in 6 can include synthesised food, as long as it can effectively help spare nature - However, in 3 fisheries can use larger areas than in 6 where MPAs are more numerous and cover a greater area |
| **Megafauna, oceans, biodiversity use** | |
| - Both 3 and 6 are based on community and traditional rights | - Dynamic protected landscapes mark out narrative 6 where humans increasingly make way for nature; - By contrast, in 3 there are no protected areas; protection of biodiversity happens uniquely through co-managing of the scape, focusing on culturally relevant species. |
| **Trade, law-rights, education, policy** | |
| - Both systems are community-based and self-reliant and apply circular economic principles. - Strong environmental education and awareness (Although more autarchic, 6 does not prevent the exchange of knowledge across long distances). | - In 3, trade is important and extensive among neighbours over some distance, while in 6 each community seeks complete self-reliance - Trade exchange in 6 is heavily based on the use of local currencies; these monetary exchange is more important than barter trade, compared to 3 where they predominate |

1. **Dynamic Natures (NN-NC or 6) vs Innovative commons (NS-NC or 4)**

| ***SIMILARITIES*** | ***DIFFERENCES*** |
| --- | --- |
| **Economy, governance, cities, communities** | |
| - Governance is polycentric with community autonomy in both 4 and 6 - Global governance is decentralised in 4 and 6 - No megacities in both 4 and 6 - Both 4 and 6 are based on the power of communities; there is community-driven exercise of local control over resources - Laws emphasise community rights, and citizens engagement in both 4 and 6 | - In 4, global governance is decentralised to the regions while in 6 it is decentralised to quasi autartic local entities. - 4 is more globalised than 6, which is more local. - In 6, cities are dynamic, flexible and adapted to nature through movement e.g., ‘nomadic cities, while in 4 cities and rural settlements are fixed and extensively connected throughout the landscape. - The system of community representation is more horizontal in 6 and more networked in 4 (more with multi-level authority). - In 6, direct interconnectivity of communities is high, while in 4, networks are stronger and connectivity with trade is higher. |
| **Infrastructure, energy, transport, water** | |
|  |  |
| **Food, diet, agriculture, fisheries, aquaculture, land management, well-being** | |
|  |  |
| **Megafauna, oceans, biodiversity use** | |
| - Both 4 and 6 are based on community and traditional rights | - Dynamic protected landscapes mark out narrative 6 where humans increasingly make way for nature; - By contrast, in 4 the amount of protected areas is limited (no more than 14%) while landscapes are widely invested by communities through social-ecological approaches. |
| **Trade, law-rights, education, policy** | |
| - Both are based on a regenerative - social economy with principles of circularity, collaboration and commoning. - Strong environmental education and awareness (Although more autarchic, 6 does not prevent the exchange of knowledge across long distances). | - In 4, trade is more extensive including trade and sharing over the Internet. |

1. **Dynamic Natures (NN-NC or 6) vs Sharing through sparing (NN-NS or 7)**

| ***SIMILARITIES*** | ***DIFFERENCES*** |
| --- | --- |
| **Economy, governance, cities, communities** | |
| - Both have distributed (i.e., not highly centralised) governance - Cities and communities are located in areas that are ecologically/geographically appropriate - Both have highly connected communities | - Governance in 6 is even more decentralised and localised, whereas in 7, main urban areas determine the scale of decentralisation (which is facilitated by AI) - CIties in 6 are more dynamic and flexible to ecological changes - Communities in 7 are more high-tech and digitally connected |
| **Infrastructure, energy, transport, water** | |
| - Novel, green infrastructure - Energy produced from renewable sources and materials, and there is an emphasis on self-sufficient communities/cities - Emphasis on air and sea travel, with new technology - Recognition of the need for hydrologic systems to be dynamic | - In 6, infrastructure is dynamic with few roads; In 7, roads remain - 6 emphasises minimising energy consumption, through innovations such as smart building practices; Future 7 emphasises innovations in renewable energy production and includes somewhat more connected cities via smart micro-grids in order to respond to shifting demand across urban landscapes - Transport in 6 captures natural forces and builds on traditional knowledge to be flexible and adaptive, whereas 7 uses other clean technologies - Water in 6 is free flowing and given legal status, whereas in 7 it is managed to provide for human society while also achieving or simulating natural flow regimes |
| **Food, diet, agriculture, fisheries, aquaculture, land management, well-being** | |
|  | - In 6, diets are diverse and seasonal - Fisheries in 6 are dominated by small-scale fisheries that are focused on sustainably supporting local communities and livelihoods, whereas in 7, fishing practices are more focused on maintaining maximal production through protection and multi-use areas, as well as globally integrated supply chains - Agriculture in 6 uses a diversity of practices, including agro-forestry, permaculture, and traditional approaches, whereas 7 focuses on sustainable maximisation of yields using high tech approaches. - 6 emphasises traditional multi-trophic eco-aquaculture systems and integration of aquaculture and agricultural systems. In 7, aquaculture emphasises productivity of lower trophic levels, and is only used in limited areas with strong environmental control and monitoring |
| **Megafauna, oceans, biodiversity use** | |
| - In both 6 and 7, there is a mixture of no-take areas and multi-use zoning and eco-tourism | - 6 uses more dynamic protected areas - In 6, species are indirectly conserved as humans make way for nature; in 7, humans take a more active role by actively stocking and reintroducing threatened species and conserving wilderness through PAs - 6 has more co-managed and co-governed fishing areas. - In 6, harvesting is more common and traditional production systems have evolved and are adapted to ecological dynamics. In 7, production is limited to a narrow number of essential species, and harvesting is permitted only through highly regulated PAs where wilderness is also conserved. |
| **Trade, law-rights, education, policy** | |
|  | - Economies in 6 are localised, non-monetary, circular, and based on local resources; In 7, there is moderate international trade, and a green economy is driven by bio- and nano-technologies. |
